# Novel GluN2B-Selective NMDA Receptor Negative Allosteric Modulator Possesses Intrinsic Analgesic Properties and Enhances Analgesia of Morphine in a Rodent Tail Flick Pain Model

**DOI:** 10.1021/acschemneuro.2c00779

**Published:** 2023-02-13

**Authors:** Lynnea D. Harris, Michael C. Regan, Scott J. Myers, Kelsey A. Nocilla, Nicholas S. Akins, Yesim A. Tahirovic, Lawrence J. Wilson, Ray Dingledine, Hiro Furukawa, Stephen F. Traynelis, Dennis C. Liotta

**Affiliations:** † Department of Chemistry, Emory University, Atlanta, Georgia30322, United States; ‡ Department of Pharmacology and Chemical Biology, Emory University, Atlanta, Georgia30322, United States; § W.M. Keck Structural Biology Laboratory, Cold Spring Harbor Laboratory, New York, New York11724, United States; ∥ RADD Pharmaceuticals, Westport, Connecticut06880, United States

**Keywords:** analgesic tolerance, opioid, morphine, NMDA receptor, subunit selectivity, tail immersion test

## Abstract

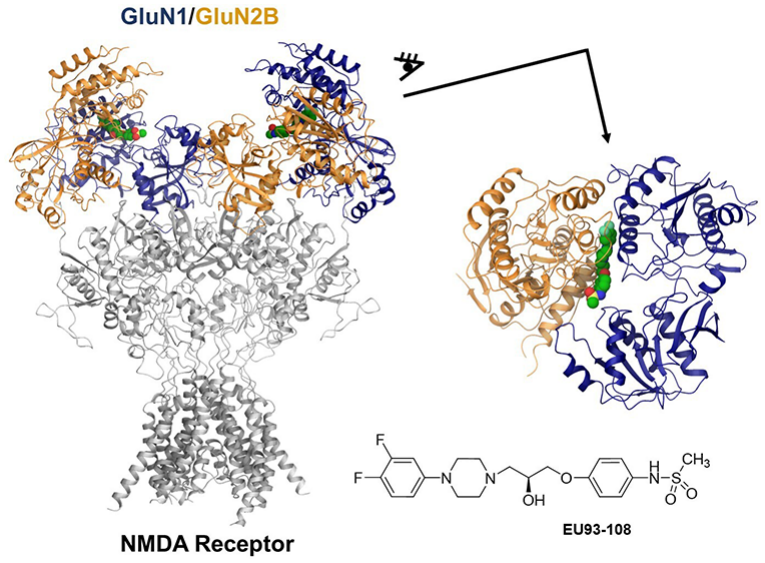

Many cases of accidental death associated with drug overdose are due to chronic opioid use, tolerance, and addiction. Analgesic tolerance is characterized by a decreased response to the analgesic effects of opioids, requiring increasingly higher doses to maintain the desired level of pain relief. Overactivation of GluN2B-containing *N*-methyl-d-Aspartate receptors is thought to play a key role in mechanisms underlying cellular adaptation that takes place in the development of analgesic tolerance. Herein, we describe a novel GluN2B-selective negative allosteric modulator, **EU93-108**, that shows high potency and brain penetrance. We describe the structural basis for binding at atomic resolution. This compound possesses intrinsic analgesic properties in the rodent tail immersion test. EU93-108 has an acute and significant anodyne effect, whereby morphine when combined with EU93-108 produces a higher tail flick latency compared to that of morphine alone. These data suggest that engagement of GluN2B as a target has utility in the treatment of pain, and EU93-108 could serve as an appropriate tool compound to interrogate this hypothesis. Future structure–activity relationship work around this scaffold could give rise to compounds that can be co-administered with opioids to diminish the onset of tolerance due to chronic opioid use, thereby modifying their utility.

## Introduction

Drug overdose is the leading cause of accidental death in the United States.^1,2^ From April 2020 to April 2021, there were over 100,000 new cases—an increase of nearly 30% compared to the previous year;^3^ 75% of these drug overdose cases involved opioids, which remain the most effective treatment available for chronic pain conditions. However, problems such as addiction, physical dependence, analgesic tolerance, and risks of overdose when abused significantly complicate their utility.^4^ Nevertheless, opioids remain important therapeutics given the crushing need for effective pain treatment. One in five people in the United States^4−6^ and globally,^7^ currently suffers from some form of chronic pain, which causes long-term disability and results in low quality of life, unemployment, anxiety, and depression.^8^ Thus, a conundrum exists whereby there is a need for drugs like opioids due to their efficacy, but different aspects of opioid actions also create problems.

Tolerance is a multifaceted phenomenon that can develop to mitigate the on-target or off-target effects of any drug.^9^ Analgesic tolerance to opioids is defined as a decreased response to the analgesic effects of opioids, such as morphine, fentanyl, oxycodone, and hydrocodone with continued use. Over time, the initial dose given becomes ineffective in relieving pain; therefore, higher doses must be used to maintain the desired level of analgesia.^10,11^ Tolerance to the analgesic effects of opioids can develop within weeks, and continually increasing doses can very quickly and unexpectedly result in fatal overdose,^12,13^ especially for people who self-administer opioids.^9,10^

**Figure 1:**
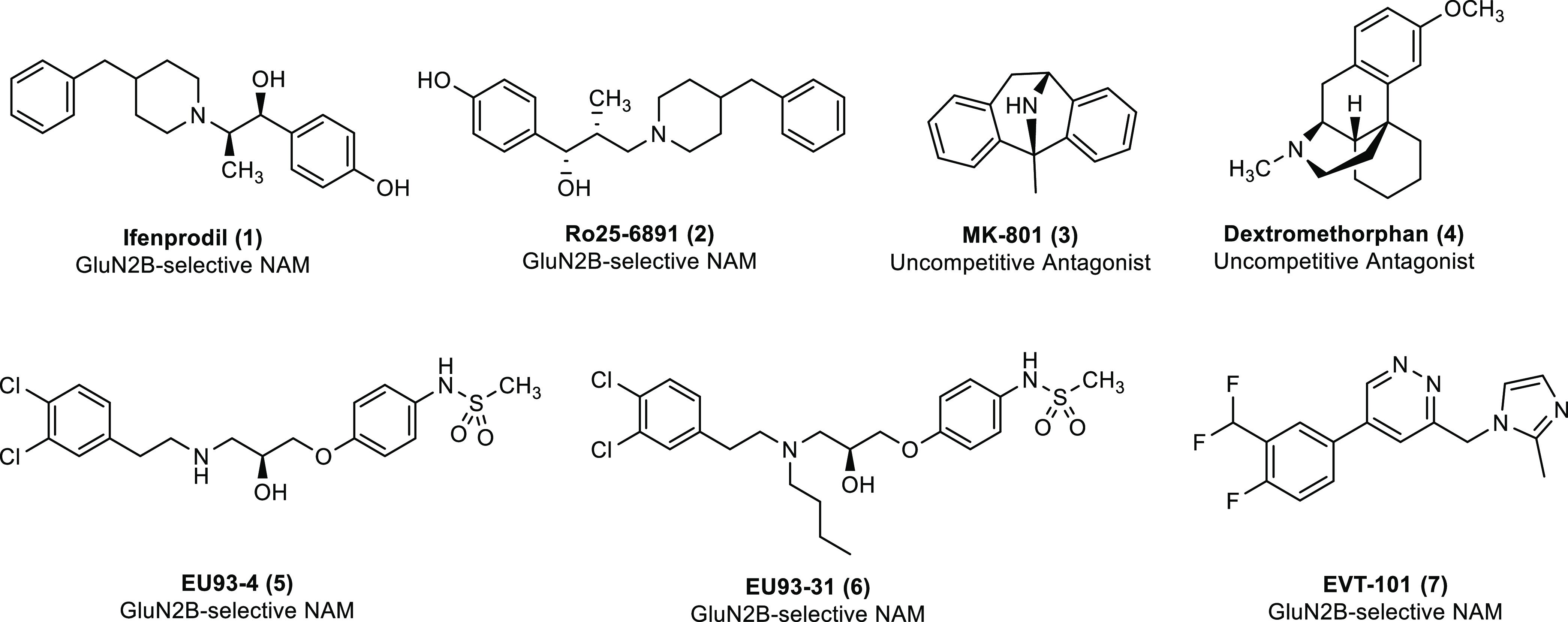
Previously published inhibitors of the NMDAR.

In the case of morphine, there are multiple cellular adaptations that contribute to the development of analgesic tolerance following chronic exposure.^14−16^ This work focuses on one specific adaptation—persistent activation of *N*-methyl-d-aspartate receptors (NMDARs) in the brain.^17−19^ NMDARs^20^ (Figure 1) are excitatory ionotropic glutamate receptors, expressed in neurons throughout the CNS, which mediate a slow Ca^2+^-permeable component of excitatory synaptic transmission, synaptic plasticity,^21,22^ learning,^23,24^ and memory.^25,26^ NMDARs are ligand-gated ion channels that are activated by the binding of the co-agonist neurotransmitters glutamate and glycine.^27^ Upon ligand binding, if the membrane becomes depolarized sufficiently to relieve Mg^2+^ block, NMDARs can pass considerable currents.^28−30^ Improper function of NMDARs has been suggested to participate in some fashion in multiple disease states such as Alzheimer’s disease,^31^ Huntington’s chorea,^32^ Parkinson’s disease,^33^ schizophrenia,^34,35^ epilepsy,^36^ ischemic brain injury,^37−39^ depression,^40,41^ and neuropathic pain.^42^

Activation of μ-opioid receptors (MORs) by opioids increases NMDAR activity via kinases PKC and Src.^17,43,44^ PKC activates Src, which phosphorylates NMDARs at the C-termini of GluN2A and GluN2B subunits, increasing the permeation of calcium into the neuron.^45,46^ This increased calcium allows for increased activation of CaMKII and nitric oxide synthase (NOS) among other signaling systems.^17,47^ CaMKII desensitizes MORs via phosphorylation,^48−50^ and NOS stimulates production of nitric oxide, which can increase glutamate release.^47,51^ This creates a cycle of sustained NMDAR activation and MOR desensitization that can contribute to analgesic tolerance development.

The GluN2B subunit is well studied in the context of neuropathic pain and analgesic tolerance because it is highly expressed throughout the nociception pathway.^52,53^ Primary afferents in the skin and tissue respond to pain and other noxious stimuli, and that information is transmitted to the spinal cord dorsal horn, specifically neurons in the substantia gelatinosa found in lamina II. The signal is then transmitted to the periaqueductal gray, thalamus, somatosensory cortex, and other regions of the brain that process painful stimuli.^54,55^ Analysis of mRNA and in situ hybridization in the CNS has shown that the dorsal horn of the spinal cord has higher mRNA levels of GluN2B compared to the other GluN2 subunits, as well as higher protein expression, which suggests that GluN2B could play a contributing role in this region.^56−58^

A large body of evidence shows that GluN2B-selective negative allosteric modulators (NAMs) including ifenprodil and Ro25-6981, and nonselective channel blockers such as MK-801 and dextromethorphan (Figure 1) can inhibit morphine tolerance in rodents.^59−61^ However, channel blockers like MK-801 and dextromethorphan are problematic for clinical use due to strong and complete block of all NMDARs and significant on-target effects. The prototypical GluN2B inhibitor, ifenprodil, has off-target actions on biogenic amine receptors, such as α-1-adrenergic receptors.^52,62^ To further evaluate and advance the idea that GluN2B inhibitors have utility in pain and can blunt tolerance, it is important to develop and characterize new compounds that will maintain efficacy in reducing tolerance while possessing an improved safety profile.

In this study, we evaluate a novel piperazine-containing GluN2B-selective NMDAR NAM^63,64^ for its effects on morphine-induced analgesic tolerance in rodents. We also assess the actions of a class of enantiomeric propanolamines that function as GluN2B-selective NAMs.^65^ Previously published compounds in this class display comparable efficacy to previous NAMs and show reduced off-target effects at concentrations up to 10x IC_50_. Compound **29** in Tahirovic et al. (referred to here as EU93-4) is brain-penetrant, neuroprotective in *in vitro* and *in vivo* models of cerebral ischemia,^65^ and did not elicit increased locomotion in rodents. We also evaluated compound **70** in Tahirovic et al., also referred to as EU93-31 (Yuan et al.).^66^ These compounds are structurally distinct from ifenprodil, but bind in the same pocket in the amino terminal domain (ATD) of the NMDAR at the interface between the GluN1 and GluN2B subunits.^67,68^ Interestingly, EU93-31 also extends the *n*-butyl chain into the pocket occupied by an unconventional GluN2B-selective inhibitor EVT-101 (Figure 1).^69^

## Results

### EU93-108 Is a Potent, GluN2B-Selective NMDAR NAM

EU93-108 is a member of a class of piperazine-containing GluN2B inhibitors that show promising properties.^63,64^ We assessed EU93-108 for its potency and subunit selectivity across NMDAR subtypes (Table 1). Two-electrode voltage clamp recordings from *Xenopus laevis* oocytes expressing recombinant rat NMDAR subunits were used to determine IC_50_ and the extent of inhibition at 10 μM concentration of EU93-108 for all NMDAR GluN2 subunits. EU93-108 was tested at 10 μM and inhibition of GluN2B was approximately 18-fold higher than that of the other NMDAR subunits, confirming that it is selective for GluN2B (Table 1).

**Table 1:**
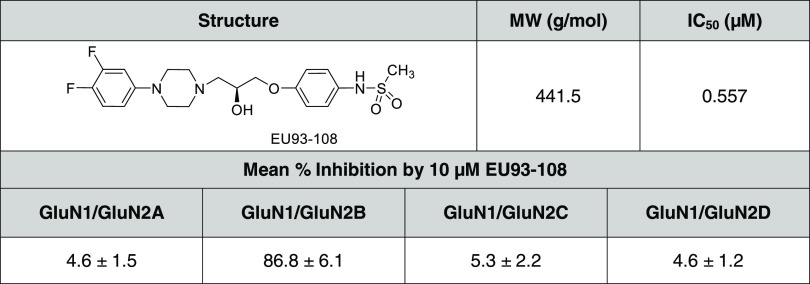
EU93-108 Is a GluN2B-Selective NMDAR NAM^a^

a The structure of EU93-108 is shown along with the molecular weight and experimentally determined IC_50_ at pH 7.4. The percent inhibition of *Xenopus* oocytes expressing recombinant GluN1/GluN2A–D receptors is presented as mean ± SEM. Oocyte experiments were performed with 10 μM EU93-108 in the presence of 100 μM glutamate and 30 μM glycine. *n* = 8 oocytes per NMDAR subtype.

### EU93-108 Concentration–Inhibition Curves on Diheteromeric and Triheteromeric NMDARs

Diheteromeric NMDARs are assembled from GluN1 and only one type of GluN2 subunit (*e.g.*, GluN1/GluN2B), and thus possess two copies of GluN1 and two copies of the same GluN2 subunit. By contrast, triheteromeric NMDARs are assembled from the GluN1 subunit and two different types of GluN2 subunits (*e.g.*, GluN1/GluN2B/GluN2A).^20^ The majority of recombinant studies have utilized diheteromeric receptors, but biochemical and functional data have shown that a large proportion of NMDARs in the CNS are triheteromeric receptors.^70,71,73^ Due to the prevalence of triheteromeric NMDARs *in vivo*, we constructed concentration–response curves for EU93-108 in both GluN2B diheteromeric and GluN2B/GluN2A triheteromeric receptors.

**Figure 2:**
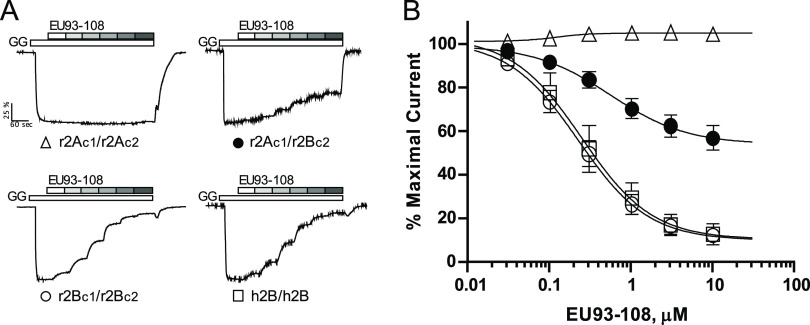
Inhibition of NMDA receptors by EU93-108. (A) Current response time course for maximal receptor activation by 100 μM l-glutamate and 100 μM glycine (GG) and then in the continuous presence of 100 μM l-glutamate and glycine plus increasing concentrations of EU93-108 at 0.03, 0.1, 0.3, 1, 3, and 10 μM is shown for each receptor subunit combination. The receptors tested are diheteromeric rat GluN1/GluN2Ac1/GluN2Ac2 (r2Ac1/r2Ac2), triheteromeric rat GluN1/GluN2Ac1/GluN2Bc2 (r2Ac1/r2Bc2), diheteromeric rat GluN1/GluN2Bc1/GluN2Bc2 (r2Bc1/r2Bc2), and diheteromeric human GluN1/GluN2B/GluN2B (h2B/h2B). All currents were normalized to the maximal response in 100 μM glutamate and glycine, set as 100%. The mean ± SEM for maximal current sizes for r2Ac1/r2Ac2 receptors was 295 ± 44 nA (*n* = 6), for r2Ac1/r2Bc2 receptors 278 ± 39 nA (*n* = 12), for r2Bc1/r2Bc2 receptors 353 ± 77 nA (*n* = 8), and for h2B/h2B receptors 276 ± 63 nA (*n* = 10). (B) Concentration–effect curve for inhibition by EU93-108 for all four receptor subunit combinations, with the same symbols as in (A). The mean ± SEM values are plotted for r2Ac1/r2Ac2 receptors (open triangles, *n* = 6), r2Ac1/r2Bc2 receptors (closed circles, *n* = 12), r2Bc1/r2Bc2 receptors (open circles, *n* = 8), and h2B/h2B receptors (open squares, *n* = 10) with increasing concentrations of EU93-108 applied in the presence of 100 μM glutamate and 100 μM glycine at −40 mV as described in the Materials and Methods section.

Concentration–inhibition curves for EU93-108 were constructed from current responses recorded from *Xenopus* oocytes expressing rat and human diheteromeric (GluN1/GluN2B/GluN2B) NMDARs, as well as from oocytes expressing rat triheteromeric (GluN1/GluN2A/GluN2B) NMDARs (Figure 2). Triheteromeric receptors contained GluN2 subunits with two coiled-coil domains (C1, C2) and an ER retention signal added to the intracellular C-terminal. The interaction of C1 and C2 can mask an exogenous ER retention signal, thereby only allowing receptors that contain one C1 and one C2 domain to be trafficked to the plasma membrane.^72^ The IC_50_ value for EU93-108 at r2Bc1/r2Bc2 receptors was 233 nM (196, 279 nM 95% CI; *n* = 8) and at r2Ac1/r2Bc2 receptors was 543 nM (460, 640 nM 95% CI; *n* = 12). The residual current remaining at 10 μM EU93-108 for r2Bc1/r2Bc2 receptors was 10.6% (7.9, 13% 95% CI; *n* = 8), and that for r2Ac1/r2Bc2 receptors was 54% (50, 58% 95% CI; *n* = 12) (Supporting Table S1; Figure 2). As anticipated, EU93-108 was also an effective inhibitor of human diheteromeric GluN2B receptors (h2B/h2B) with an IC_50_ of 260 nM (197, 347 nM 95% CI; *n* = 10) and with a residual current remaining at 10 μM EU93-108 of 12% (8.5, 16% 95%CI; *n* = 10). Finally, EU93-108 shows no appreciable activity at r2Ac1/r2Ac2 diheteromers exhibiting a residual current at 10 μM of 105% (102, 108% 95% CI; *n* = 6) (Supporting Table S1; Figure 2).

Taken together, these data demonstrate substantial selectivity for inhibition of GluN2B versus GluN2A NMDA receptors. In addition, EU93-108 is more potent and can achieve a greater degree of maximal receptor inhibition in 2B/2B diheteromeric assemblies compared to 2A/2B triheteromeric assemblies, thus potency and efficacy increase as the number of copies of the GluN2B subunit increases from one to two copies per receptor complex. Both IC_50_ potency and maximum % inhibition of r2Ac1/r2Bc2 receptors were significantly different from r2Bc1/r2Bc2 and h2B/h2B receptors by a one-way analysis of variance (ANOVA) and Tukey’s multiple comparison test, *p* < 0.05 or better. We also compared concentration–inhibition curves for rat and human 2B/2B assemblies, which show that EU93-108 inhibits rat and human 2B diheteromeric receptors in a similar manner.

An important control for triheteromeric experiments is to confirm that a minimal proportion of receptors contain two copies of GluN2A or two copies of GluN2B. To confirm the negligible contribution from diheteromeric receptors, we introduced two mutations (R518K, T690I for GluN2A and R519K, T691I for GluN2B, referred to as R-K,T-I) into the binding pockets of the GluN2 subunits. By injecting mRNA encoding one C-tagged subunit and the other C-tagged GluN2 subunit with the R-K,T-I mutations functional currents will only be expressed if diheteromeric receptors containing two copies of the subunit lacking the mutations escape the ER and reach the cell surface. By comparing the current amplitude in this situation to that observed for functional triheteromeric receptors, we can estimate the percentage of current that is from diheteromeric receptors (Supporting Figure S1).

### Crystal Structure of GluN1b-GluN2B ATD in Complex with EU93-108

**Figure 3:**
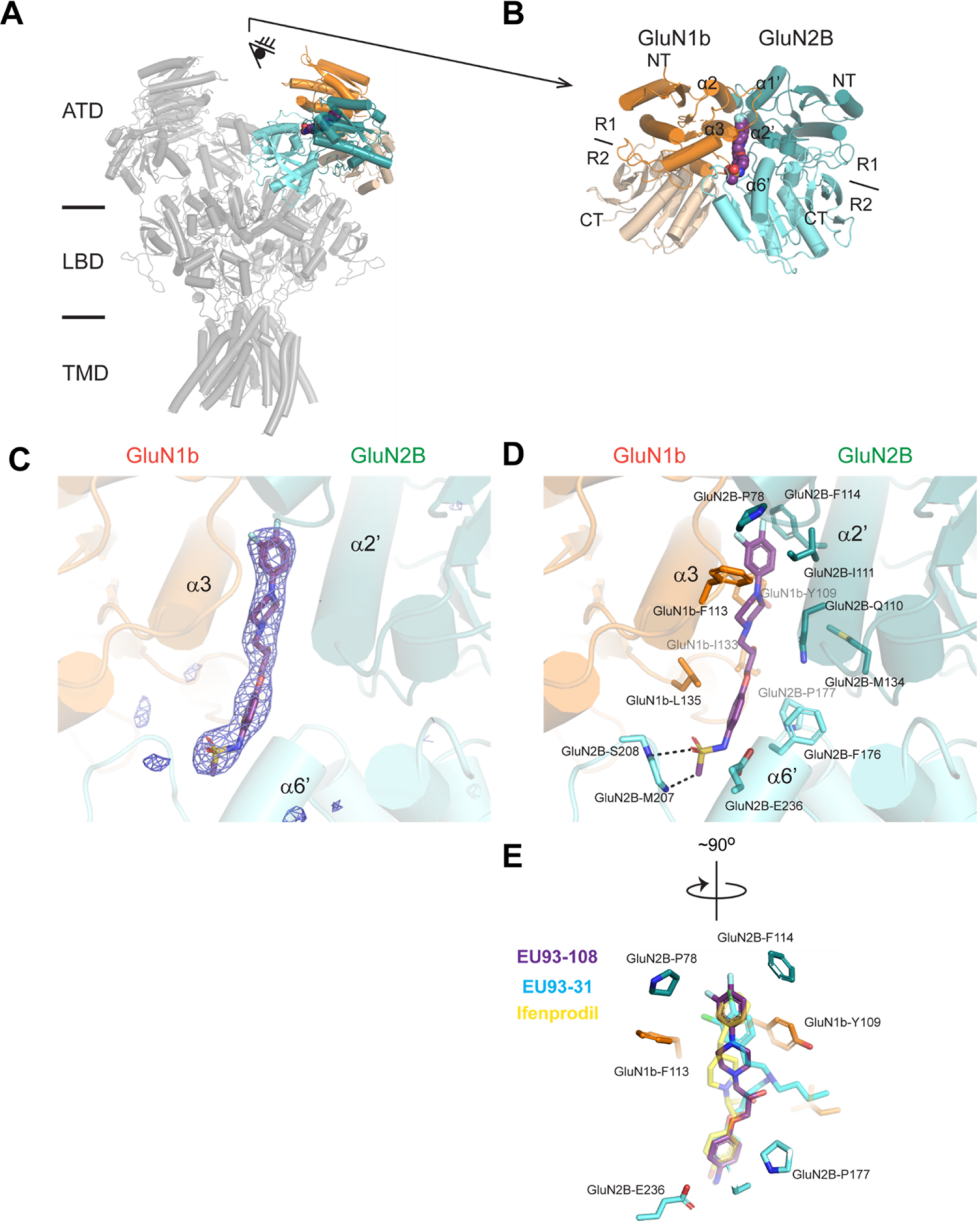
Structure of GluN1b-GluN2B ATD in complex with EU93-108. (A) GluN1b-GluN2B ATD bound to EU93-108 is superposed to the structure of the intact GluN1b-2B NMDAR in complex with glycine and glutamate in non-active1 (PDB code: 7SAA; in gray). GluN1b-R1, GluN1b-R2, GluN2B-R1, and GluN2B-R2 are colored dark orange, light orange, dark cyan, and light cyan, respectively. (B) GluN1b-GluN2B ATD viewed from the eye in (A). EU93-108 is shown as spheres. (C) FoFc omit map contoured at σ = 3.8. (D) Binding site of EU93-108 (purple stick). The interacting residues are shown as sticks. Dash represents polar interaction. (E) Superposition of EU93-108 with ifenprodil (yellow; PDB: 3QEL), EU93-31 (cyan; PDB: 6E7U).

Once we established that EU93-108 is GluN2B-selective, we next wanted to confirm that this compound binds at the ATD interface of GluN1b and GluN2B, in the same pocket as ifenprodil, EU93-31, and other previously published GluN2B-selective NAMs.^67,68^ To ascertain the binding site and pose for EU93-108, we utilized protein crystallography and X-ray diffraction to solve the structure of the isolated GluN1b-GluN2B ATD in complex with EU93-108 at 2.85 Å resolution (Figure 3, Supporting Table S2). It has been well established that the structure of the isolated ATDs is identical to the ATDs of the intact tetrameric receptors; thus, the EU93-108-bound structure presented here is physiologically relevant (Figure 3A,B).^68,74−76^ Specifically, the GluN1b-GluN2B ATD-EU93-108 structure is similar to the non-active1 conformation of the intact GluN1b-2B NMDARs (RMSD *vs*7SAA = 1.964 Å over 662 Cαs), where the bi-lobe structure (composed of R1 and R2) of GluN2B ATD is closed.^77−79^ The quality of the electron density is sufficient for identifying and modeling EU93-108 with confidence (Figure 3C), which permits us to visualize the binding mode precisely (Figure 3D).

The crystal structure revealed the binding site of EU93-108 at the GluN1b-GluN2B ATD heterodimer interface, which is similar to that of EU93-31 and ifenprodil. Specifically, the binding site involves residues from GluN1b and GluN2B ATDs, especially around the α3 helix from GluN1b and α2′ and α6′ from GluN2B. The sulfonamide group of EU93-108 forms polar interactions with the backbone amides of GluN2B-Met207 and -Ser208. The phenyl group, the piperazine group, and the difluorophenyl group are in van der Walls contacts with residues such as GluN2B-Pro78, -Phe176, -Pro177, -Ile111, and -Phe114 and GluN1-Phe113, -Ile133, and -Leu135. EU93-108 has similar sets of polar interactions as EU93-31^67^ but not ifenprodil^75^ (Figure 3E). The van der Walls contacts are similar between EU93-108, ifenprodil, and the backbone of EU93-31 (Figure 3E).

### Determination of Intrinsic Antinociceptive Properties of GluN2B-Selective NAMs

**Figure 4:**
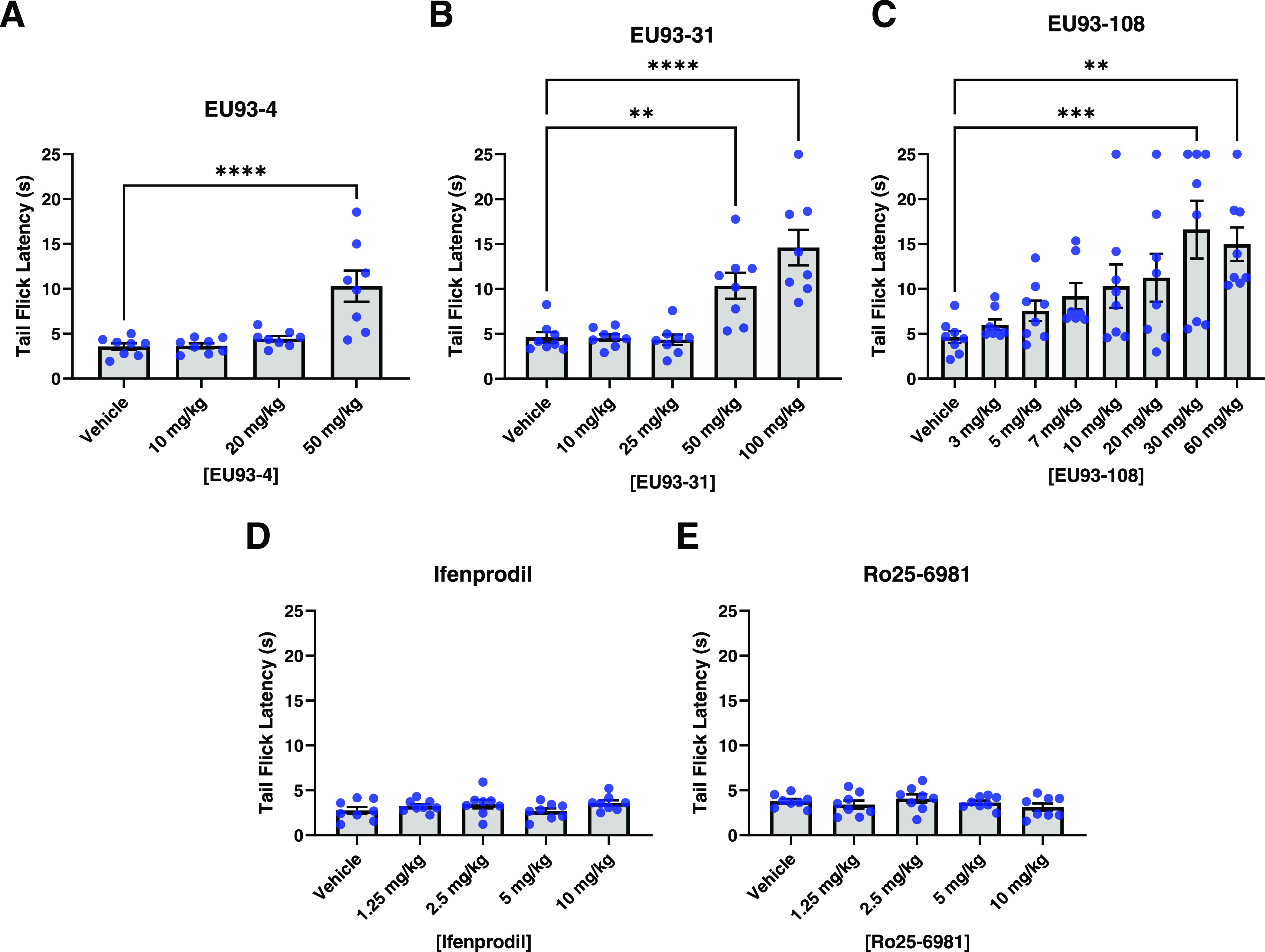
Intrinsic antinociceptive properties of (A) EU93-4, (B) EU93-31, (C) EU93-108, (D) ifenprodil, and (E) Ro25-6981 in male C57BL/6J mice (hot water tail immersion test). Each dot represents one mouse (*n* = 8 per group), and data are presented as mean ± SEM. Data were analyzed using one-way ANOVA and Dunnet’s *post hoc* test for multiple comparisons, where each group was compared to vehicle.

**Figure 5:**
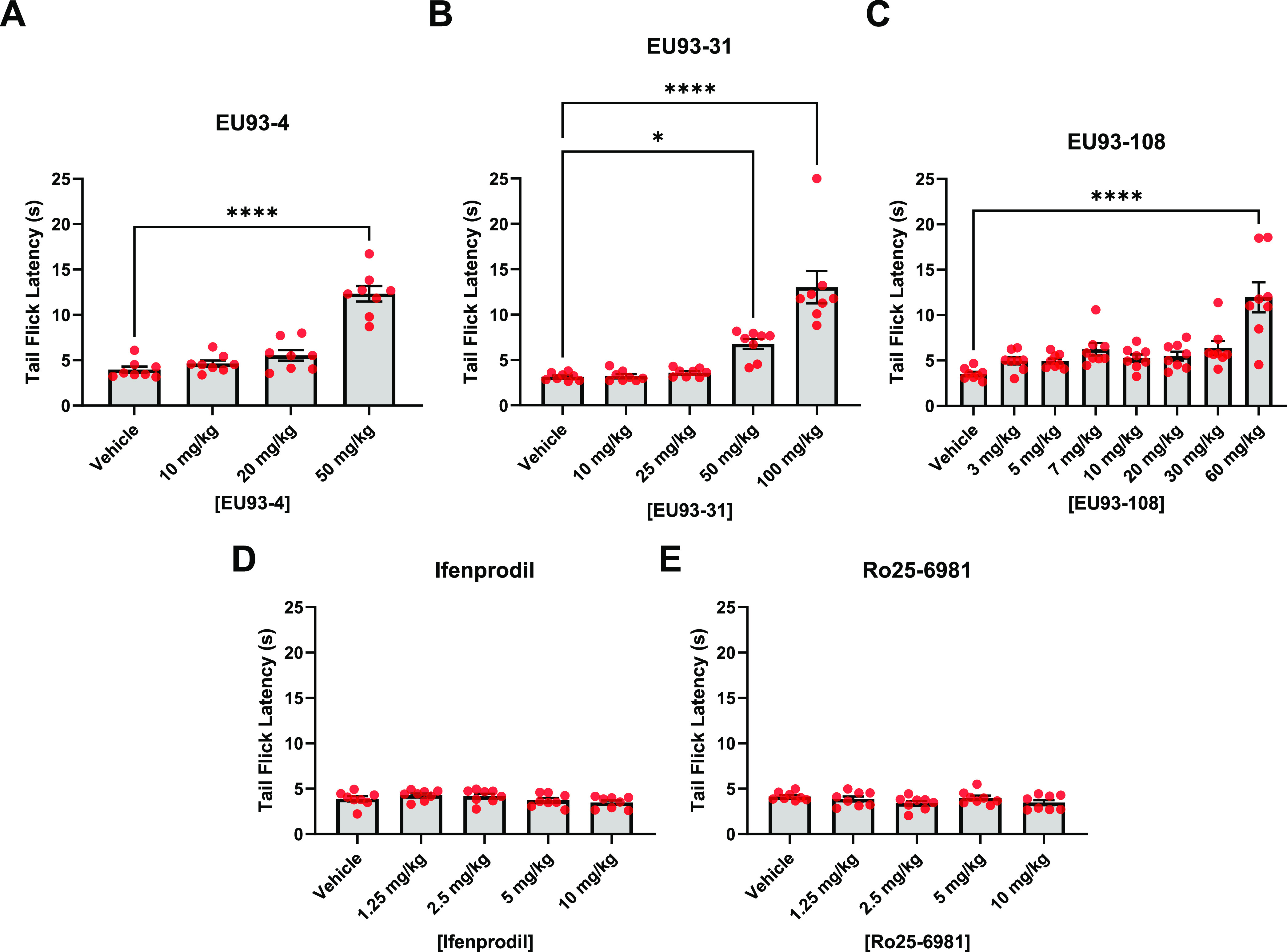
Intrinsic antinociceptive properties of (A) EU93-4, (B) EU93-31, (C) EU93-108, (D) ifenprodil, and (E) Ro25-6981 in female C57BL/6J mice. Each dot represents one mouse (*n* = 8 per group), and data are presented as mean ± SEM. Data were analyzed using one-way ANOVA and Dunnet’s *post hoc* test for multiple comparisons, where each group was compared to vehicle.

After evaluating EU93-108 *in vitro*, we next evaluated a panel of GluN2B inhibitors for their ability to produce an antinociceptive effect using a classical model of pain perception, determination of tail flick latency for mice when their tails are placed in hot water (Figures 4 and 5). The hot water tail immersion test is a well-validated method of assessing reflexive (*i.e.*, spinal) pain-like response in rodents.^80−83^ We interpret a drug-induced increase in tail flick latency as analgesia.

The inhibitors were injected i.p. 30 min prior to a hot water tail immersion test. Four treatment groups and one control group were used (*n* = 8 per group) where the control group received vehicle (10% DMSO, 20% PEG, 2% DMA in water) and groups 1 through 4 were randomly assigned one dose of the appropriate compound.

Male mice that received compound EU93-4 displayed significant increases in tail flick latency at 50 mg/kg, the highest dose tested (Figure 4). Mice that received EU93-31 displayed significant increases in latency at the two highest doses tested (50 and 100 mg/kg). Mice that received EU93-108 also displayed significant increases in latency at the two highest doses tested (30 and 60 mg/kg). The mean latency increase in the EU93-108 group was significantly higher than that of the EU93-4 or EU93-31 groups—16 seconds compared to 10 seconds. Male mice that received ifenprodil or Ro25-6981 did not display significant increases in latency at the doses tested. These data suggest that EU93-108 has more potent intrinsic analgesic effects than the other inhibitors tested. Significant latency increases were achieved at a lower dose of EU93-108 compared to EU93-4 or EU93-31 (30 mg/kg compared to 50 mg/kg). Similar analgesic effects were found for 93-108 in the Chung spinal nerve ligation model of allodynia (Supporting Figure S2).

Female mice that received EU93-4 or EU93-31 displayed significant increases in latency at the highest doses tested for each compound, 50 and 100 mg/kg, respectively (Figure 5). Female mice that received EU93-108 also displayed increased latency, but only at 60 mg/kg, the highest dose tested. The mean increase of this group was comparable to the EU93-4 and EU93-31 groups. As seen in the male mice, ifenprodil and Ro25-6981 did not produce any significant increases in latency at the doses tested. EU93-4 and EU93-31 have similar intrinsic analgesia in male and female mice. However, EU93-108 appears to be more potent in male mice because a significant increase in latency was achieved at half the dose in males compared to females (30 mg/kg compared to 60 mg/kg).

### EU93-108 has Sustained Brain and Plasma Concentrations

Given the direct effect of EU93-108 in the tail immersion test, we explored its pharmacokinetic properties by measuring brain and plasma concentrations over the course of 4 h following an i.p. injection. In male Sprague–Dawley rats, brain and plasma concentrations of EU93-108 were measured 30, 120, and 240 min following an i.p. injection of 60 mg/kg.

**Figure 6:**
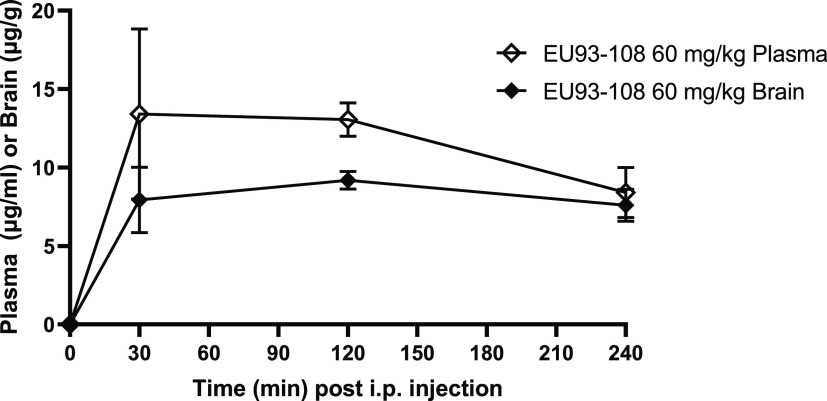
EU93-108 has sustained plasma and brain concentrations over 4 h. EU93-108 concentrations are shown for plasma (open diamonds) and brain (closed diamonds). Brain and plasma concentrations were measured at 0, 30, 120, and 240 min post injection. Each symbol indicates *n* = 3 rats. All injections were given i.p. and results are shown as mean ± SEM.

Brain concentrations were measured from whole forebrain homogenate at each time point. EU93-108 reached peak concentration 30 min post injection which was sustained for the duration of the experiment. EU93-108 reached a *C*_max_ of 18 μM in brain and 30 μM in plasma after 60 min, yielding a brain-to-plasma ratio of 0.6. Due to the minimal decrease in concentration over the course of the 240 min experiment, the half-life of EU93-108 must be greater than 4 h (Figure 6).

### Acute Morphine and EU93-108 Co-Administration Produces Enhanced Tail Flick Latency

After observing that EU93-108 possessed intrinsic antinociceptive properties in the hot water tail immersion test, we chose to focus on this compound for the remaining experiments. We were next interested in evaluating the effects of EU93-108 when given acutely in combination with morphine. 5 mg/kg morphine was chosen because this dose elicited an approximately half-maximal response in our initial morphine dose–response curve (Supporting Figure S3). This half-maximal response would allow us to see any changes in tail flick latency that EU93-108 might elicit.

Four treatment groups and one control group were used (*n* = 8 per group). The control group received vehicle (10% DMSO, 20% PEG, 2% DMA in water) i.p. plus 5 mg/kg morphine s.c. formulated in normal saline, and groups 1–4 were randomly assigned one dose of EU93-108 i.p. plus 5 mg/kg morphine s.c. The doses used were the same as in the intrinsic analgesia experiments.

**Figure 7:**
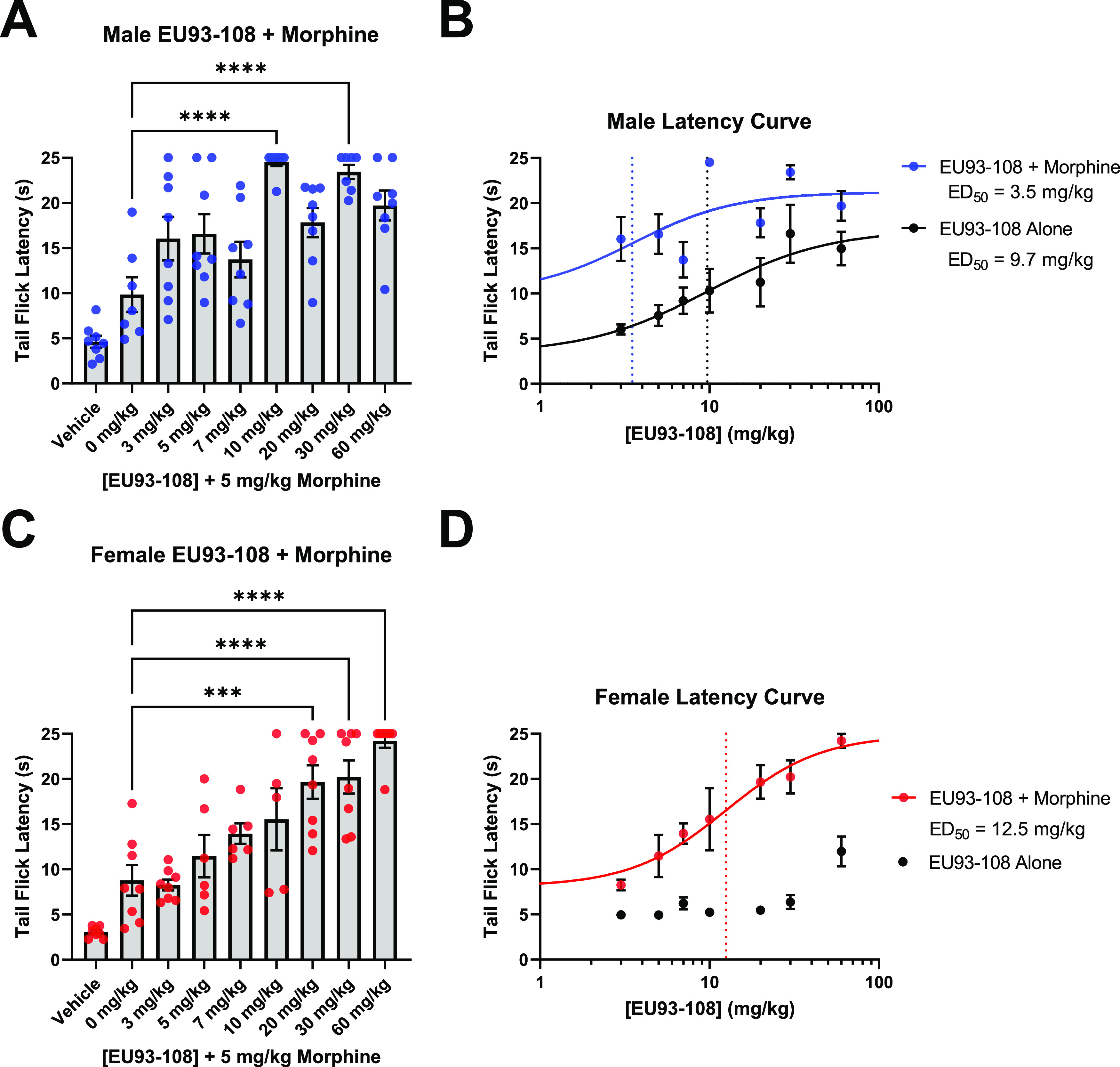
(A, C) Acute co-administration of EU93-108 and 5 mg/kg morphine in male (A, blue circles) and female (C, red circles) mice. Each dot in (A) and (C) represents one mouse (*n* = 8 per group). Data were analyzed using one-way ANOVA and Tukey’s *post hoc* test for multiple comparisons, where all groups were compared to each other. (B, D) Fitted dose–response curves for EU93-108 in male (B) and female (D) mice. The dotted lines depict estimated ED_50_ values. All data are presented as mean ± SEM.

A dose-dependent increase in tail flick latency was observed in both male and female mice. In male mice, the highest tail flick latency was observed when 10 mg/kg EU93-108 was combined with 5 mg/kg morphine (Figure 7A). The majority of mice in this group did not have a tail flick response within 25 s in three consecutive tests (*i.e.*, maximal analgesia). 10 mg/kg was the lowest dose that significantly increased latency in male mice. Figure 7B depicts the fitted ED_50_ curves for EU93-108 with and without morphine in male mice. In male mice, EU93-108 is approximately 3-fold more potent when combined with morphine versus EU93-108 alone (Figure 7B). In female mice, the lowest dose of EU93-108 that significantly increased latency was 20 mg/kg (Figure 7C). The effect of EU93-108 in female mice was less variable than male mice, and apparently more robust. However, the estimated ED_50_ for EU93-108 plus morphine in female mice was higher than in male mice, suggesting that lower doses of EU93-108 may be more potent in male mice compared to female mice. The data for EU93-108 alone in female mice did not yield a sufficient curve and was therefore not fitted (Figure 7D).

### EU93-108 Has Sedative Effects at High Doses

Some NMDAR antagonists can impact locomotor behavior in animals.^84−86^ Lower doses have minimal impact while high doses can have anesthetic action that can produce complete immobility. We evaluated this potential side effect in a locomotor assay using a range of doses of EU93-108. We used these data along with the previously described data in Figures 4 and 5 to choose a target dose to use for the chronic morphine administration/tolerance experiments shown in Figures 10 and 12. We defined a target dose as one that exhibits enhancement in tail flick latency when combined with morphine, while showing little to no effect on locomotor activity.

**Figure 8:**
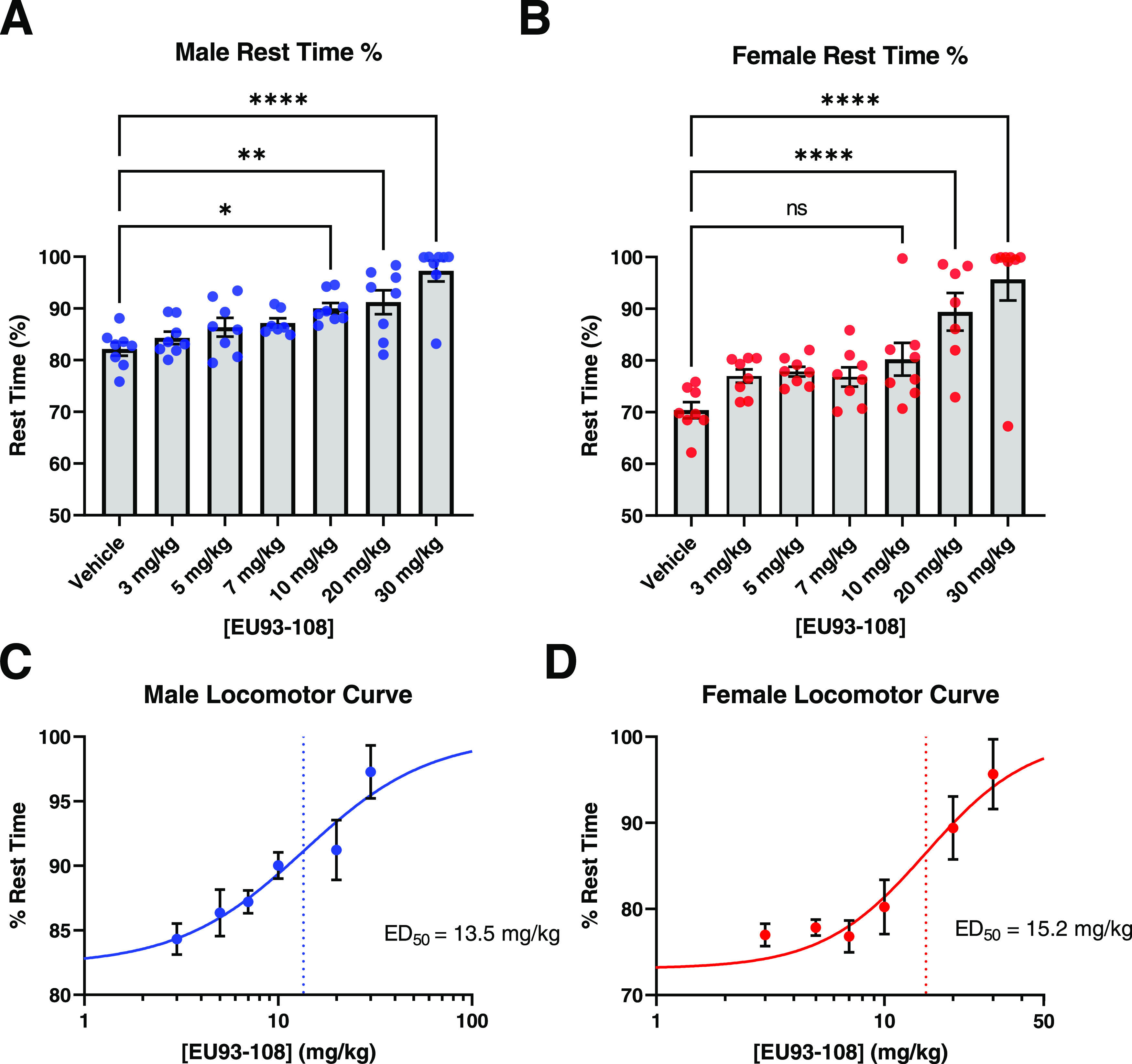
Rest time data for EU93-108 in male (A, blue circles) and female (B, red circles) mice. Rest time percentage was used to calculate dose–response curves for males and females ((C) and (D), respectively). Each circle in (A) and (B) represents one mouse (*n* = 8 per group), while each circle in (C) and (D) represents the mean ± SEM of all 8 mice for each group. The dotted lines depict estimated ED_50_ values. Data were analyzed using one-way ANOVA and Dunnet’s *post hoc* test for multiple comparisons, where each group was compared to vehicle.

In both males and females, we observed dose-dependent decreases in total distance traveled, number of movements made, and percentage of time spent moving (Supporting Figure S4). Conversely, we observed a dose-dependent increase in percentage rest time (Figure 8A,B). Groups that received 30 mg/kg EU93-108 showed sedation, with rest time increasing from 72% to on average 99% of the experiment time. We used rest time percentage to calculate the sedation ED_50_ curves shown in Figure 8C,D. ED_50_ values were similar for males and females (Figure 8C,D).

### Chronic Co-Administration of Morphine with EU93-108 Did Not Inhibit the Development of Tolerance

Given that acute doses of EU93-108 increase tail flick latency in the presence and absence of morphine, we subsequently assessed changes in tail flick latency when given chronically (*i.e.*, multiple injections over multiple days). Specifically, we were interested in whether the presence of EU93-108 would inhibit the development of analgesic tolerance to morphine.

To decide on a target dose for the chronic administration studies, we used the difference between our tail flick latency ED_50_ and locomotor activity ED_50_ as the therapeutic window between increase in tail flick latency (*i.e.*, analgesia) and sedation. For males, the ED_50_ of analgesia with morphine was 3.5 mg/kg (Figure 7) and the ED_50_ of sedation was 13.5 mg/kg (Figure 8). We chose 10 mg/kg EU93-108 because that dose falls within the therapeutic window, it yielded maximal analgesia with morphine, and it is below the ED_50_ of the sedation curve. We know from our previous data that females require higher doses to achieve the same effects seen in males. In our tail flick data, we saw that females required twice the dose needed for males, so we selected 20 mg/kg EU93-108 for testing in female mice.

**Figure 9:**
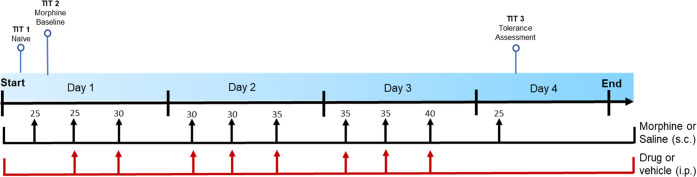
Morphine analgesic tolerance “stair stepping” dosing regimen. TIT = tail immersion test. On day 1, TIT was conducted to determine baseline latencies for all mice. On days 1 through 3, each mouse was administered morphine s.c. three times per day at doses increasing from 25 to 40 mg/kg at 8:00 am, 12:00 pm, and 4:00 pm. Doses of morphine are shown above the arrows, in mg/kg. The mice were also randomly assigned to receive vehicle, one dose of EU93-108 (10 mg/kg for males and 20 mg/kg for females), or one dose of ifenprodil (10 mg/kg for males and 20 mg/kg for females), i.p. at the same time points. Each mouse received an i.p. injection of drug or vehicle followed by an s.c. injection of morphine or saline. On day 4, each mouse was challenged with the minimum dose of morphine (25 mg/kg) 30 min prior to a tail immersion test.

To assess whether EU93-108 had an inhibitory effect on the development of morphine tolerance, four groups were used (*n* = 8 per group): vehicle/saline, vehicle/morphine, ifenprodil/morphine, and EU93-108/morphine. Morphine tolerance was induced using repeated administration of increasing doses of morphine from 25 to 40 mg/kg three times a day at 3 to 4 h intervals over three consecutive days (Figure 9). This procedure was adapted from previous publications.^87−89^

**Figure 10:**
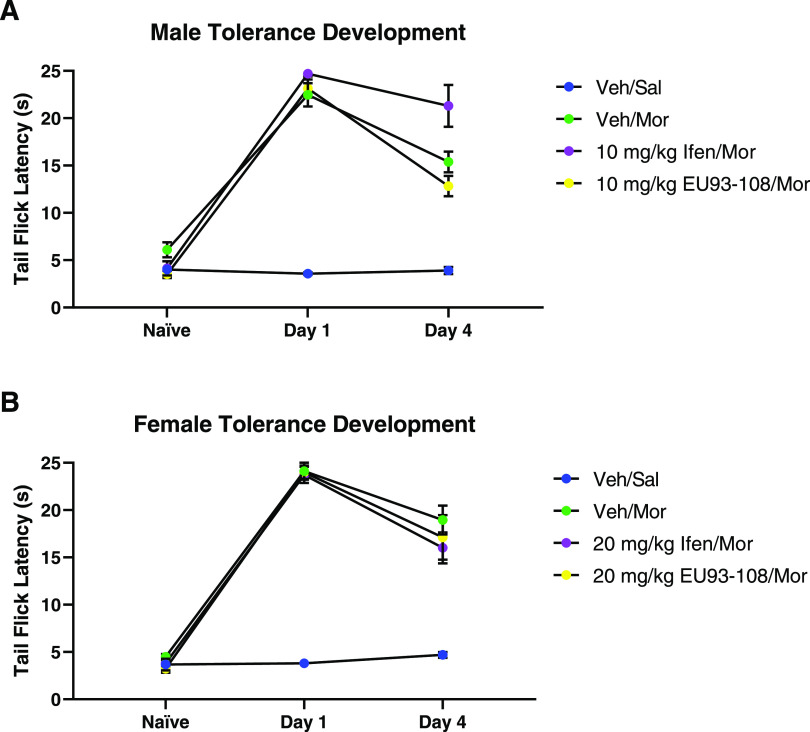
Effects of co-administration of EU93-108 and morphine on tolerance development in (A) male and (B) female mice. Mice received vehicle and saline (blue circles), vehicle and morphine (green circles), ifenprodil and morphine (purple circles), or EU93-108 and morphine (yellow circles). Naïve indicates latencies prior to the first morphine dose. Day 1 indicates latencies after first morphine dose, and day 4 indicates latencies after final morphine dose once tolerance has developed. Each circle represents *n* = 8 mice, and data are presented as mean ± SEM.

The vehicle/morphine groups, which were the tolerance controls, successfully developed a tolerance phenotype, as shown by the significant decrease in latency on day 4 compared to day 1 (Table 2). In both male and female experiments, EU93-108 failed to inhibit the development of tolerance when co-administered with morphine three times a day for three consecutive days (Figure 10).

**Table 2:** *P* Values for Tolerance Development Experiments (Figure 9)^a^

comparison	paired or unpaired?	unadjusted *p* value	Bonferroni-corrected p value	significant after adjustment?
Male Mice				
day 4 108/mor - day 1 108/mor	paired	0.0004	0.0016	yes
day 4 veh/mor - day 1 veh/mor	paired	0.0043	0.0172	yes
day 4 ifen/mor - day 1 ifen/mor	paired	0.139	0.558	no
day 4: 108/mor - veh/mor	unpaired	0.118	0.47	no
Female Mice				
day 4 108/mor - day 1 108/mor	paired	0.0317	0.127	no
day 4 veh/mor - day 1 veh/mor	paired	0.0156	0.0624	no
day 4 ifen/mor - day 1 ifen/mor	paired	0.0003	0.0012	yes
day 4: 108/mor - veh/mor	unpaired	0.553	2.21	no

a For male and female mice, all relevant comparisons were analyzed using paired or unpaired Student’s *t*-tests as appropriate. *t*-tests were followed by Bonferroni correction where each *p*-value was multiplied by the total number of comparisons made, yielding an adjusted *p*-value. We also indicated whether the adjusted *p*-values were significant (<0.05).

### Co-administration of EU93-108 and Morphine Slows Worsening of Preestablished Tolerance

**Figure 11:**
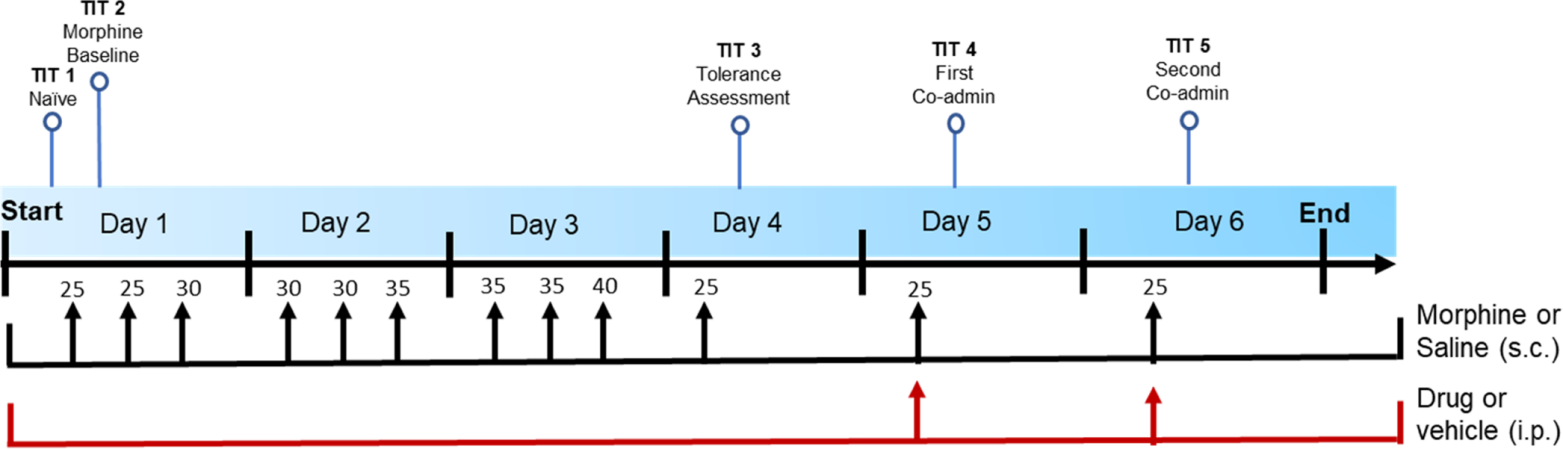
Dosing regimen to assess effects of EU93-108 on tolerant mice. TIT = tail immersion test. All mice received morphine or saline three times a day according to the protocol in Figure 9. Doses of morphine are shown above the arrows, in mg/kg. Once tolerance was established on day 4, the mice were randomly assigned to receive vehicle, EU93-108, or ifenprodil once a day for two consecutive days. TIT was conducted at the same time points as in Figure 9 and 30 min after injection on days 5 and 6.

**Figure 12:**
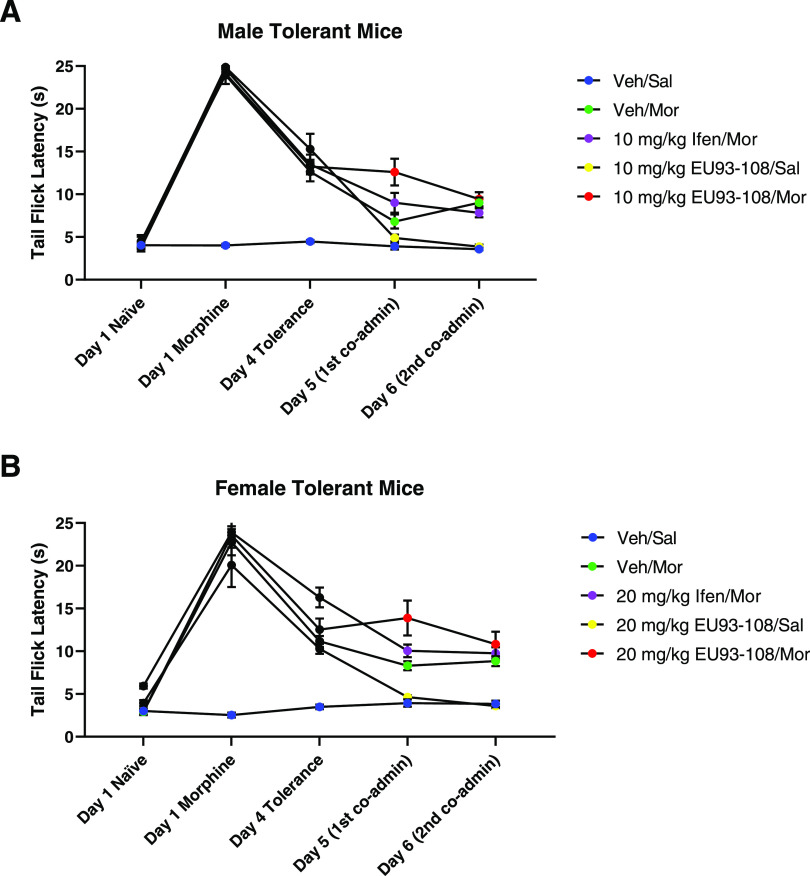
Effects of EU93-108 on tolerant (A) male and (B) female mice. Black circles show that all mice were treated the same and given only morphine to establish tolerance on days 1 through 4. Once tolerance was established (day 4 Tolerance), the mice were randomly assigned to receive vehicle and morphine (green circles), ifenprodil and morphine (purple circles), EU93-108 and saline (yellow circles), or EU93-108 and morphine (red circles). Blue circles depict mice that did not receive any drug for the duration of the experiment. For the male mice, the dose of ifenprodil and EU93-108 was 10 mg/kg, and for female mice, the dose was 20 mg/kg. Days 1 through 4 depict tolerance development. Days 5 and 6 represent latencies 30 min after the first and second co-administrations, respectively.

We next wanted to assess EU93-108 for effects on other facets of tolerance. Specifically, we were interested in whether EU93-108 could increase tail flick latency in mice that were already tolerant to morphine (*i.e.*, preestablished tolerance). To assess the effect of EU93-108 on preestablished tolerance, five groups were used (*n* = 8 per group): vehicle/saline, vehicle/morphine, ifenprodil/morphine, EU93-108/saline, and EU93-108/morphine. All groups except for the vehicle/saline control group were administered morphine three times per day (according to the stair stepping protocol outlined in Figure 11) at gradually increasing doses from 25 to 40 mg/kg three times a day at 3–4 h intervals for three consecutive days until tolerance was observed on day 4 (Figure 12, black circles).

Once tolerance was established, mice were randomly assigned to receive vehicle, EU93-108, or ifenprodil once a day for two consecutive days (Figure 12). The doses differed between males and females: males received 10 mg/kg EU93-108 and 10 mg/kg ifenprodil, while females received 20 mg/kg EU93-108 and 20 mg/kg ifenprodil. Tail immersion tests were conducted 30 min post injection of EU93-108 or ifenprodil on days 5 and 6.

Male and female mice in all groups successfully developed tolerance over days 1 through 4 (Figure 12, black circles). The vehicle/morphine, ifenprodil/morphine, and EU93-108/saline latencies all continued to decrease on day 5 (Table 3), followed by a plateau on day 6. The EU93-108/saline group showed the most significant decrease in latency and plateaued at values equivalent to the vehicle/saline baseline. In contrast, the EU93-108/morphine group did not show a further decrease in latency on day 5, meaning the latencies on days 4 and 5 were equivalent. The EU93-108/morphine group also had the highest latency on day 5 compared to the other groups. In the male mice, this increase in latency was only seen on day 5, whereas in females the effect remained constant through day 6. Morphine alone and EU93-108 alone did not increase tail flick latency in tolerant mice, but EU93-108 plus morphine did increase latency. This suggests that EU93-108 requires co-administration with morphine to slow the worsening of the tolerance phenotype in tolerant mice.

**Table 3:** *p* Values for Tolerant Mice Experiments (Figure 12)^a^

comparison	paired or unpaired?	unadjusted *p* value	Bonferroni-corrected *p* value	significant after adjustment?
Male Mice				
day 4 108/Sal - day 5 108/Sal	paired	0.0024	0.012	yes
day 4 108/Mor - day 5 108/Mor	paired	0.711	3.55	no
day 4 Veh/Mor - day 5 Veh/Mor	paired	0.0252	0.126	no
day 5: 108/Mor - 108/Sal	unpaired	0.0004	0.002	yes
day 5: 108/Mor - Veh/Mor	unpaired	0.0268	0.134	no
Female Mice				
day 4 108/Sal - day 5 108/Sal	paired	0.0002	0.001	yes
day 4 108/Mor - day 5 108/Mor	paired	0.543	2.72	no
day 4 Veh/Mor - day 5 Veh/Mor	paired	0.0035	0.0175	yes
day 5: 108/Mor - 108/Sal	unpaired	0.0003	0.0015	yes
day 5: 108/Mor - Veh/Mor	unpaired	0.0146	0.073	no

a All relevant comparisons were analyzed using paired or unpaired Student’s *t*-tests as appropriate. *t*-tests were followed by Bonferroni correction where each *p*-value was multiplied by the total number of comparisons made, yielding an adjusted *p*-value. We also indicated whether the adjusted *p*-values were significant (<0.05).

### Off-Target Effects of EU93-108

EU93-108 was further examined for its behavior against common off-target receptors. First, selectivity for GluN2B over other ion channels in the brain was examined via two-electrode voltage clamp recordings of *Xenopus* oocytes expressing AMPA, kainate, nicotinic acetylcholine, serotonin, GABA, glycine, and ATP receptors. Current responses were tested with saturating concentrations of agonist in both the absence and presence of 10 μM EU93-108, and confirmed selectivity for GluN2B-containing NMDA receptors over AMPA, kainate, GABA, glycine, ATP, and 5-HT_3A_ receptors (Supporting Table S3).

Compound EU93-108 was also tested for inhibition of binding of probes to a range of GPCRs and other targets (Table 4 and Supporting Table S4). We found that EU93-108 has multiple off-target receptor interactions, some of which are consistent with liabilities of previously described GluN2B-selective NAMs including ifenprodil.^66,90−94^ EU93-108 produced significant displacement of binding probes for the 5-HT_2_ receptors and α-1-adrenergic receptors, as well as D_3_, H_1_, and σ_1_ receptors. Concentration–response binding competition curves for these targets were used to determine K_i_ values (Table 4). Given that the brain concentration of EU93-108 achieved 30 min post i.p. injection is 18 μM (Figure 6), the doses that yield our desired antinociceptive effects may engage some of the receptors listed in Table 4.

**Table 4:** Secondary Off-Target Screen of EU93-108^a^

receptor	log(*K*_i_)	*K*_i_ (nM)	*K*_i_/IC_50_ of EU93-108	receptor	log(*K*_i_)	*K*_i_ (nM)	*K*_i_/IC_50_ of EU93-108
5-HT_2A_	–6.31	490	0.88	α_2B_	–6.14	728	1.31
5-HT_2B_	–6.01	976	1.75	α_2C_	–6.06	862	1.55
5-HT_2C_	–5.74	1825	3.28	D_3_	–6.72	190	0.34
α_1A_	–6.77	170	0.31	H_1_	–6.59	254	0.46
α_1D_	–6.44	359	0.65	σ_1_	–6.3	505	0.91
α_2A_	–5.94	1151	2.07				

a Concentration–response curves were constructed for any receptors that showed 50% or greater mean inhibition in a primary high-throughput screen (Supporting Table S3). The associated *K*_i_ values are shown along with the ratio of *K*_i_ to the IC_50_ for EU93-108 (555 nM). Ratios less than 1 correspond to receptors for which EU93-108 has a higher affinity compared to GluN2B-containing NMDARs. These constitute the strongest off-target effects.

## Discussion

The 93 series is a class of potent, brain-penetrant, GluN2B-selective NAMs. These compounds have shown utility as *in vitro* and *in vivo* tool compounds but have never been evaluated in the context of pain and opioid tolerance. We report a novel and potent GluN2B-selective NMDAR inhibitor, EU93-108, and explore the structural basis for its binding to the GluN1/GluN2B NMDAR ATD using X-ray crystallography. This compound is highly brain-penetrant and maintains high brain and plasma concentrations for at least 4 h post i.p. injection. EU93-108 possesses intrinsic analgesic properties in the rodent tail immersion test. We also observed a significant, acute enhancement in tail flick latency where the combination of EU93-108 and morphine yielded higher latencies compared to either compound alone. This combination also transiently slowed worsening of tolerance in tolerant mice.

Limitations of EU93-108 include several off-target interactions, some of which have previously been described as liabilities for other GluN2B-selective NAMs. The strongest interactions were at α-1-adrenergic, D_3_, H_1_, and 5-HT_2_ receptors. Among the interactions observed, the sedative effect seen in the locomotor data may reflect inhibition of the H_1_ histamine receptor and could complicate the use of this compound as a tool for *in vivo* experiments.

The favorable effects of EU93-108 appear to be acute as opposed to chronic or cumulative. The most promising data shown in this study corresponds to the immediate effects of EU93-108 at *T*_max_ (30 min). In the tolerance development experiments shown in Figure 10, we did not observe any cumulative effect of multiple doses of EU93-108 when the mice were assessed for tolerance.

Another interesting aspect of EU93-108 is that it has opposite effects in tolerant versus nontolerant mice when given alone, whereas with morphine, it has similar acute effects in either case. In Figures 3–5, we showed that EU93-108 alone can increase analgesia in nontolerant mice. However, once mice have developed tolerance to morphine, administering EU93-108 without morphine appears to further exacerbate tolerance (Figure 12, yellow circles). When EU93-108 is co-administered with morphine, we observed enhancement of morphine-induced analgesia in nontolerant mice, as well as an acute plateau of tolerance in tolerant mice. In both cases, the combination of EU93-108 and morphine yielded favorable effects.

The potency of EU93-108 is sex-dependent, with males requiring lower doses than females for the same effect. Two potential explanations for this sexual dimorphism that have been quantified in the literature are differences in CYP expression and differences in analgesia. It is well documented that male and female mice have differential expression of several CYP enzymes.^95−97^ For example, CYP2D9 is almost exclusively expressed in male mice,^95^ whereas CYPs 2B9 and 2B13 are almost exclusively expressed in female mice.^95^ CYP3A4, the isoform responsible for approximately 50% of phase-I metabolism of drugs, shows higher expression and activity in female mice.^98^ This could suggest that EU93-108 is cleared faster in female mice and therefore more frequent dosing might be needed to see increased analgesic effects. CYP reaction phenotyping of EU93-108 would be needed to explore this idea further.

Pain tolerance and analgesia differ between strains of mice^99^ and between sexes.^99−103^ Female mice tend to have lower pain tolerance than males.^99,100,104^ Opioids such as morphine also have higher potency in male mice compared to female mice.^100,101,105^ This suggests that female mice might require higher doses of opioids compared to male mice. Additionally, morphine is metabolized faster in female mice.^106^ This is primarily due to the increased activity of UGT2B7, the primary UDP-glucuronosyltransferase that metabolizes morphine into its two main metabolites, M3G and M6G.^107−109^ This suggests that elimination of drugs is accelerated in female mice, therefore more frequent dosing of opioids or other analgesics might be required to achieve the same level of pain relief observed in males.

This work introduces a promising tool compound on which to base future SAR studies. The insights gained from EU93-108 help to create a blueprint for the next generation of GluN2B-selective inhibitors, highlighting aspects of GluN2B negative modulation that are beneficial and some that are detrimental in the context of pain relief. We have demonstrated that negative modulation of GluN2B can both increase analgesia in the absence of an opioid and enhance the analgesic properties of an opioid with co-administration. These are aspects that need to be maintained in the next generation of compounds based on this scaffold. Conversely, EU93-108 has a number of off-target liabilities, therefore the next iteration of inhibitors must possess an improved off-target profile. Ultimately, this new generation of GluN2B-selective NAMs may be evaluated for clinical use alongside opioids. These candidates would be co-administered with opioids to enhance their effect, which would decrease the dose of opioid needed for suitable analgesia. Decreasing opioid dose could decrease the rate of development of tolerance and decrease risk of physical dependence and addiction with chronic use.

## Materials and Methods

### Chemicals

Buffers, salts, agonists, and ifenprodil-(±)-tartrate salt were purchased from Millipore Sigma. Morphine sulfate was purchased from McKesson Medical Surgical. All other compounds were synthesized at Emory according to published methods or as described below. Ifenprodil was formulated in 10% DMSO, 20% PEG, 2% DMA in water. Morphine sulfate was formulated in a 0.9% saline solution. All 93 series compounds were formulated in 10% DMSO, 20% PEG, and 2% DMA in water.

### General 93 Series Synthesis^65^

**Scheme 1:**
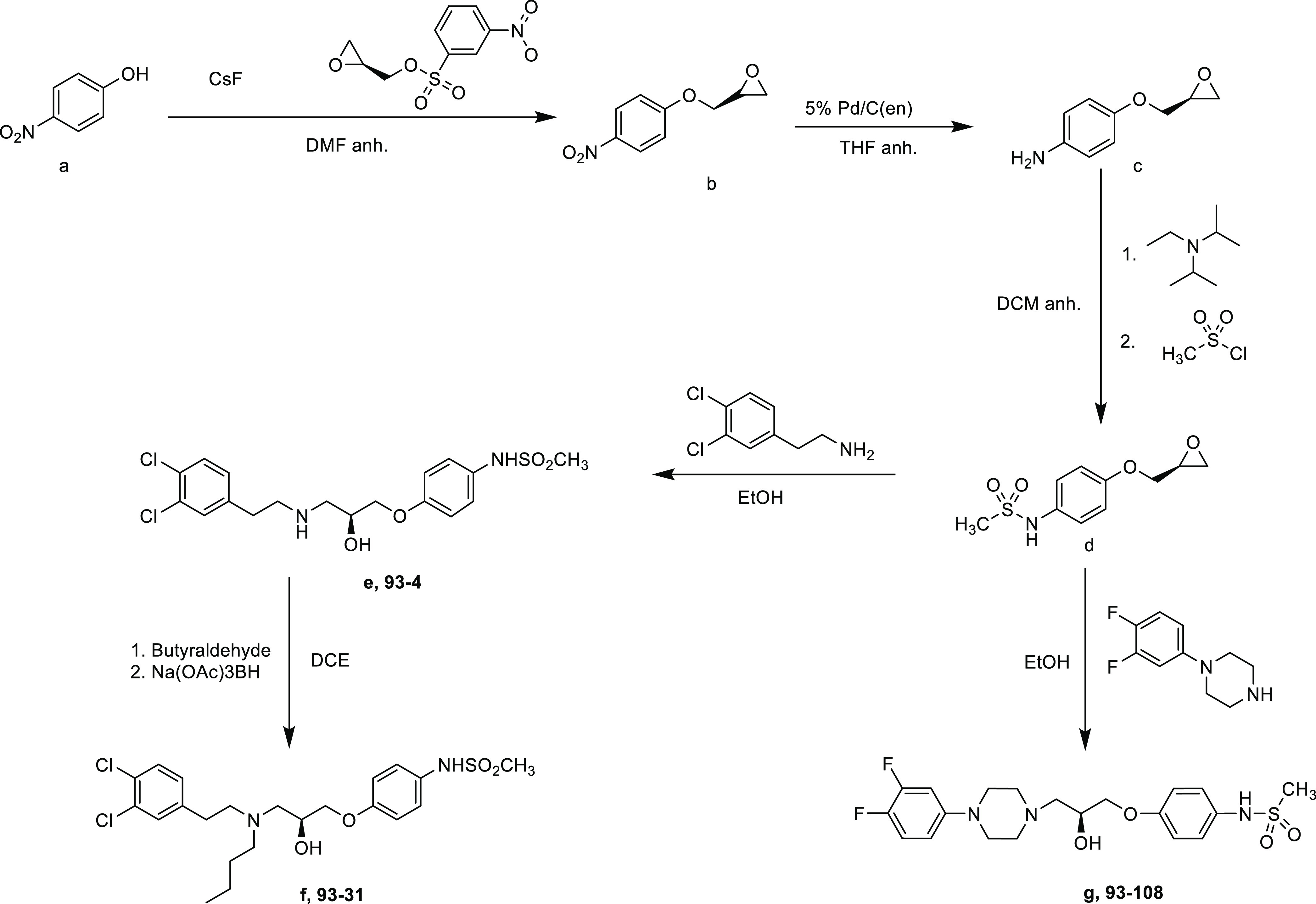
93 Series Synthesis *para*-Nitrophenol was combined with (*S*)-(±)-glycidyl nosylate and cesium fluoride to afford the nitro intermediate. (b) The nitro group was reduced to an amine using poisoned palladium on carbon. The unstable amine was immediately combined with *N*,*N*-diisopropyl-*N*-ethyl amine and methane sulfonylchloride to afford the sulfonamide intermediate. (d) The sulfonamide was combined with 3,4-dichlorophenethylamine under reflux conditions to afford EU93-4. EU93-31 was afforded by combining EU93-4 with the appropriate aldehyde and sodium triacetoxyborohydride. EU93-108 was synthesized by combining the previous sulfonamide intermediate, (d) and 1-(3,4-difluorophenyl)piperazine in ethanol under reflux conditions.

The compounds EU93-4 and EU93-31 (Scheme 1) were synthesized according to previously published methods.^65,110,111^

### Synthesis of EU93-108 ((*S*)-*N*-(4-(3-(4-(3,4-Difluorophenyl)piperazin-1-yl)-2-hydroxypropoxy)phenyl)methanesulfonamide)

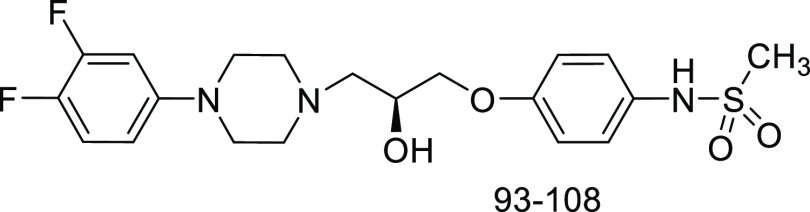



*N*-[4-[[(2S)-Oxiran-2-yl]methoxy]phenyl]methanesulfonamide (75 mg, 0.31 mmol) and 1-(3,4-difluorophenyl)piperazine (61 mg, 0.31 mmol) were dissolved in ethanol (5 mL) and heated at reflux for 3–4 h. The reaction mixture was then cooled to rt, and the solvent was evaporated in vacuo. The remaining residue was then purified via column chromatography on silica gel using 0–30% 90:10:0.5% DCM/methanol/NH_3_ in DCM to yield  *N*-[4-[(2*S*)-3-[4-(3,4-difluorophenyl)piperazin-1-yl]-2-hydroxy-propoxy]phenyl]methanesulfonamide (82 mg, 0.19 mmol in 60% yield).

^1^H NMR (500 MHz, CDCl_3_): δ 7.20-–7.12 (m, 2H), 7.02 (dt, *J* = 10.3, 9.2 Hz, 1H), 6.91–6.85 (m, 2H), 6.69 (ddd, *J* = 13.3, 6.8, 2.9 Hz, 1H), 6.60–6.53 (m, 1H), 4.15–4.09 (m, 1H), 4.00–3.92 (m, 2H), 3.15–3.10 (m, 4H), 2.92–2.89 (m, 3H), 2.84–2.77 (m, 2H), 2.68–2.59 (m, 3H), 2.56 (dd, *J* = 12.5, 3.8 Hz, 1H). ^13^C NMR (126 MHz, CDCl_3_): δ 156.96, 150.24, 148.20, 144.35, 129.74, 124.34, 115.45, 111.53, 109.98, 105.44, 70.49, 65.67, 60.40, 53.13, 49.54, 38.76. HRMS calcd for C_26_H_26_O_4_N_3_F_2_S 442.16066; found 442.16148 [M + H].

### Two-Electrode Voltage Clamp Recordings from *X. laevis* Oocytes

Rat cDNA encoding GluN1-1a, (GluN1, RefSeq NP_058706), GluN2A (NP_036705), GluN2B (NP_036706), GluN2C (NP_036707), and GluN2D (NP_073634) were obtained from Drs. S. Heinemann (Salk Institute), S. Nakanishi (Kyoto University), and P. Seeburg (University of Heidelberg). cRNA was transcribed in vitro from linearized plasmids containing NMDAR cDNAs according to the manufacturer’s instructions (mMessage mMachine, Ambion; Thermo Fisher Scientific, Waltham, MA). NMDARs were expressed in *X. laevis* oocytes following microinjection of 3–5 ng of the GluN1 subunit cRNA and 7–10 ng of GluN2B subunit cRNA in 50 nL of RNAse free water as previously described.^112^ Oocytes were incubated in Barth’s solution at 18 °C, and recordings were made 2–7 days after the injections at room temperature using two two-electrode voltage clamp amplifiers at a holding potential of −40 mV.

Oocytes were perfused with a solution of 90 mM NaCl, 1 mM KCl, 10 mM HEPES, and 0.5 mM BaCl_2_, and the pH was adjusted to 7.4 using 1 M NaOH. 10 μM of EDTA was added to chelate contaminant divalent ions such as Zn^2+^. Oocytes were placed in a dual-track plexiglass recording chamber that was assumed to be at a reference potential of 0 mV. The glass microelectrodes were filled with 300 mM KCl for the voltage electrode, and 3 M KCl for the current electrode. Bath clamps communicating across silver chloride wires were placed into each side of the recording chamber. The IC_50_ data was obtained by applying 100 μM glutamate and 30 μM glycine, followed by the application of glutamate and glycine plus increasing concentrations of the test compound up to 30 μM. Current responses of less than 50 nA were not included. The level of inhibition was calculated as a percent of the initial glutamate response, averaged across all oocytes from a single frog. Each experiment used 6–7 oocytes from the same frog. The results from these experiments were pooled and fitted to the equation,
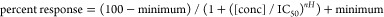
where minimum is the residual percent response at saturating concentration of the test compound and *nH* is a slope factor for the steepness of the inhibition curve.

### Triheteromeric NMDAR Constructs

Triheteromeric receptor constructs were generated using rat GluN1 and GluN2A with modified C-terminal peptide tags as previously described.^72^ Briefly, C-terminal peptide tags were generated from leucine zipper motifs found in GABA_B1_ (referred to as C1) and GABA_B2_ (referred to as C2). These tags were placed downstream of a synthetic helical linker and upstream of a KKTN endoplasmic reticulum retention signal.^113−115^ The tag was introduced in frame and in place of the stop codon at the GluN2A C-terminal tail to make 2A_C1_ and 2A_C2_. A chimeric GluN2B subunit was constructed in which the 2B carboxyl tail after residue 838 was replaced by the GluN2A carboxyl tail and C-terminal-linker-C1 or -C2-ER retention motifs to make 2B_AC1_ and 2B_AC2_.^72^ The C1 and C2 leucine zipper motifs can form a coiled-coil structure that masks the KKTN retention motif and allows for expression of only triheteromeric receptors on the cell surface. Recordings were taken at pH 7.4.

Measurement of “escape” currents was used to assess the efficiency of the peptide tags which control surface expression. Our average escape currents were typically less than 10% and this was considered an acceptable threshold. Currents were estimated using pairs of mutations (GluN2A-R518K,T690I and GluN2B-R519K,T691I) that render the agonist binding domain incapable of binding glutamate, and therefore unable to pass current.

### Expression and Purification of Intact NMDARs

Expression and purification methods of intact NMDAR receptors were based on previously established methods.^79^ The membrane fractions (100 mg/mL) of the infected insect cells were solubilized in the buffer containing 20 mM HEPES–Na pH 7.5, 150 mM NaCl, 1 mM glycine, 1 mM Na-glutamate, and 0.5% lauryl maltose neopentyl glycol (LMNG) for 2 h at 4 °C and centrifuged at 125,000*g* for 40 min. The supernatant was purified using Strep-Tactin resin followed by Superose 6 Increase column (GE Healthcare) size exclusion chromatography (SEC), which was preequilibrated with 20 mM HEPES–Na pH 7.5 and 150 mM NaCl. All of the purification steps above were conducted in the absence of glycine and glutamate.

### Structural Biology of GluN1b-GluN2B ATD

Coexpression and purification of the *X. laevis* GluN1b and rat GluN2B ATD heterodimer were performed as described previously.^74,75^ Briefly, Trichoplusia ni (High Five, Thermo Fisher) insect cells were infected with a baculovirus harboring *Xenopus* GluN1b ATD and rat GluN2B ATD cDNAs for 48 h. The concentrated medium was subjected to purification by Chelating-Sepharose charged with CoCl_2_. Poly-histidine tags at the C-terminus of GluN1b ATD and the N-terminus of the GluN2B ATD were removed by thrombin digestion and the digested samples were further purified by Superdex200 (GE Lifescience). Purified protein was concentrated to 10 mg/mL and dialyzed against 50 mM NaCl, 10 mM Tris (pH 8.0), and 1 μM ifenprodil hemi-tartrate (Tocris). The dialyzed protein was filtered through a 0.1 μm spin filter (Millipore) prior to the crystal screens. Crystals grew in sodium formate/HEPES as previously described,^74^ taking 3–4 days to appear, then continuing to grow for up to 2–3 weeks at 18 °C. Crystals were transferred to 2 μL drops containing 4 M sodium formate, 0.1 M HEPES (pH 7.5), 35 mM NaCl, 7 mM Tris (pH 8.0), and 50 μM of EU93-108, and allowed to soak overnight. Crystals were then transferred to a new drop of the same condition and soaked overnight again. Crystals were flash-frozen in liquid nitrogen for X-ray diffraction data collection by sequentially transferring them to 4.5 M and 5 M sodium formate and left overnight.

### Determination of Plasma and Brain Concentrations of EU93-108

EU93-108 was formulated in 2% dimethylacetamide (DMA), 10% dimethyl sulfoxide (DMSO), and 20% PEG in sterile water and administered intraperitoneally (i.p.) with a 10 mL/kg dose volume to adult male Sprague–Dawley rats (7–8 weeks, approx. 200 g; Charles River). At 30, 120, and 240 min post administration the rats were briefly anesthetized with isoflurane and then decapitated. Trunk blood was collected in K-EDTA tubes and then spun in a microcentrifuge at 3500 rpm for 10 min to separate plasma, which was then transferred to a clean tube and frozen on dry ice. To prepare forebrain tissue samples, the whole brain was removed from the skull, the cerebellum and brainstem cut away, the meninges were removed, and the forebrain was then rinsed with ice-cold normal saline, after which it was blotted dry with filter paper and weighed on a microbalance. To each forebrain 2.5 mL of ice-cold 50 mM K-phosphate buffer (pH 7.4) was then added and the samples homogenized with a hand-held homogenizer. The brain homogenates were then transferred to clean tubes (2 per brain) and frozen on dry ice.

Plasma and brain homogenate samples were analyzed by LC-MS/MS operating in multiple reaction monitor mode (MRM) by Ricerca Biosciences (Dublin, OH). Briefly, plasma and brain were centrifuged at 4000 rpm for 15 min to clarify a supernatant from which fractions were collected and injected onto the LC-MS/MS. The amount of parent compound in each plasma or brain sample was calculated by comparing the response of the analyte in the sample to that of a standard curve (Ricerca, OH).

### Hot Water Tail Immersion Paradigm^118−121^

All animal studies performed at Emory have been approved by the Institutional Animal Care and Use Committee at Emory University. Male and female C57BL/6J mice weighing between 20 and 30 g were housed five per cage and given *ad libitum* access to food and water. The mice were 8–10 weeks of age at the time of experimentation. Animal holding rooms operated on a 12 h dark/light cycle with the dark cycle from 7:00 pm to 7:00 am. Mice were allowed to acclimate in cages for one week after arrival. The mice were handled regularly and habituated to scruffing and cloth restraint for 3–5 days prior to experimentation.

Mouse weights were recorded on the day of each experiment. Mice were given the appropriate treatment(s) 30 min prior to testing. All injection volumes were 10 mL/kg (*e.g.*, 350 μL for a 35 g mouse). For the tail immersion experiments, each mouse was restrained with a Wypall cloth, leaving the tail exposed. The distal two-thirds of the tail was lowered into a sous vide water bath set to 48 °C, and a stopwatch with a resolution of 0.01 s was used to record the time elapsed between immersion and tail flick. Three recordings were taken for each mouse, and each data point is expressed as the mean of the three recordings. A cut-off time of 25 s was implemented to prevent tissue damage or scarring. For each experiment, the identities of the doses were coded by an independent researcher to ensure blinding. The identities were not decoded until the data were analyzed.

Prism 9.3.1 software (GraphPad, San Diego, CA) was used for all data analysis and visualization. Data for intrinsic antinociception was analyzed using one-way ANOVA and Dunnett’s *post hoc* test for multiple comparisons where each group was compared to vehicle. Data for acute morphine potentiation experiments were analyzed using one-way ANOVA and Tukey’s *post hoc* test for multiple comparisons was also used, where each group mean was compared to the mean of every other group. Data for tolerance experiments were analyzed using repeated measures two-way ANOVA and Bonferroni *post hoc* analysis. Data are presented as mean ± SEM and *p* < 0.05 constitutes significance.

An *a priori* power analysis was conducted using G*Power3^122^ to test the difference between two independent group means using a two-tailed test, a large effect size (*d* = 0.80), and an α of 0.05. The results showed that a total sample of *n* = 7 animals per group was required to achieve a power of 0.80.

### Locomotor Assessment

For both male and female mice, *n* = 8 mice per group were used. Locomotor activity was assessed at 3, 5, 7, 10, 20, and 30 mg/kg EU93-108. Mice were brought to the experiment room the night before and given *ad libitum* access to food and water. Each mouse was given an i.p. injection of the appropriate dose or vehicle 30 min prior to starting the locomotor boxes (Versamax420 Animal Activity Monitoring System, AccuScan Instruments, Inc., Columbus, OH). Movements of the mice were tracked for 1 h then the mice were placed back in their cages. Data were analyzed using one-way ANOVA and Dunnett’s *post hoc* test for multiple comparisons where each group was compared to vehicle. Data are presented as mean ± SEM.

### Radioligand Binding Assay

Conventional competition and saturation radioligand binding assays were used to determine the affinities of reference standards and EU93-108. Experiments were carried out by the NIMH Psychoactive Drug Screening Program (PDSP) and were performed as previously described.^123^

The detailed experimental protocols for the radioligand assays are available on the NIMH PDSP website at https://pdsp.unc.edu/pdspweb/content/UNC-CH%20Protocol%20Book.pdf.

## Supporting Information Available

Table of EU93-108 concentration–inhibition results at NMDA receptors expressed in oocytes (Table S1); mean oocyte escape current in response to 100 μM glutamate and 100 μM glycine (Figure S1); X-ray crystallographic data collection and model refinement statistics (Table S2); mechanical allodynia data for EU93-108 performed in male Sprague–Dawley rats (Figure S2); morphine dose–response curve in male C57BL/6J mice (Figure S3); locomotor activity of EU93-108 (Figure S4); off-target actions of EU93-108 at ligand-gated ion channels expressed in oocytes (Table S3); and off-target actions of EU93-108 on the panel of GPCR targets (Table S4) (PDF)

## References

[ref1] Rudolph K. E.; Kinnard K. E.; et al. The Relative Economy and Drug Overdose Deaths. Epidemiology 2020, 31, 551 10.1097/EDE.0000000000001199. 32332222 PMC7505523

[ref2] Rudd R. A.; Seth P.; David F.; Scholl L. Increases in drug and opioid-involved overdose deaths — United States, 2010-2015. Morb. Mortal. Wkly. Rep. 2016, 65, 1445–1452. 10.15585/mmwr.mm655051e1. 28033313

[ref3] Niles J. K.; Gudin J.; Radcliff J.; Kaufman H. W. The Opioid Epidemic Within the COVID-19 Pandemic: Drug Testing in 2020. Popul. Health Manag. 2021, 24, S43–S51. 10.1089/pop.2020.0230. 33031013 PMC7875135

[ref4] Dahlhamer J.; Lucas J.; et al. Prevalence of Chronic Pain and High-Impact Chronic Pain Among Adults — United States, 2016. Morb. Mortal. Wkly. Rep. 2018, 67, 1001–1006. 10.15585/mmwr.mm6736a2. PMC6146950 30212442

[ref5] Yong R. J.; Mullins P. M.; Bhattacharyya N. Prevalence of chronic pain among adults in the United States. Pain 2022, 163, E328–E332. 10.1097/j.pain.0000000000002291. 33990113

[ref6] Kennedy J.; Roll J. M.; Schraudner T.; Murphy S.; McPherson S. Prevalence of Persistent Pain in the U.S. Adult Population: New Data From the 2010 National Health Interview Survey. J. Pain 2014, 15, 979–984. 10.1016/j.jpain.2014.05.009. 25267013

[ref7] Goldberg D. S.; McGee S. J. Pain as a global public health priority. BMC Public Health 2011, 11, 770 10.1186/1471-2458-11-770. 21978149 PMC3201926

[ref8] Lawrence A.; Kaul A.; Seaver M. Chronic Pain 5-Minute Clin. Consult Stand. 2016 Twenty Fourth Ed. 2021, 10.1542/9781581109689-part01-ch40.

[ref9] Ling G. S. F.; Paul D.; Simantov R.; Pasternak G. W. Differential development of acute tolerance to analgesia, respiratory depression, gastrointestinal transit and hormone release in a morphine infusion model. Life Sci. 1989, 45, 1627–1636. 10.1016/0024-3205(89)90272-5. 2555641

[ref10] Morgan M. M.; Christie M. J. Analysis of opioid efficacy, tolerance, addiction and dependence from cell culture to human. Br. J. Pharmacol. 2011, 164, 1322–1334. 10.1111/j.1476-5381.2011.01335.x. 21434879 PMC3229764

[ref11] Buntin-Mushock C.; Phillip L.; Moriyama K.; Palmer P. P. Age-dependent opioid escalation in chronic pain patients. Anesth. Analg. 2005, 100, 1740–1745. 10.1213/01.ANE.0000152191.29311.9B. 15920207

[ref12] Schiller E. Y.; Goyal A.; Mechanic O. J. Opioid Overdose. Challenging Cases Complicat. Manag. Pain Med. 2022, 3–7. 10.1007/978-3-319-60072-7_1.

[ref13] White J. M.; Irvine R. J. Mechanisms of fatal opioid overdose. Addiction 1999, 94, 961–972. 10.1046/j.1360-0443.1999.9479612.x. 10707430

[ref14] Williams J. T.; Ingram S. L.; et al. Regulation of m-Opioid Receptors: Desensitization, Phosphorylation, Internalization, and Tolerance. Pharmacol. Rev. 2013, 65, 223–254. 10.1124/pr.112.005942. 23321159 PMC3565916

[ref15] Ingram S. L.; Macey T. A.; Fossum E. N.; Morgan M. M. Tolerance to repeated morphine administration is associated with increased potency of opioid agonists. Neuropsychopharmacology 2008, 33, 2494–2504. 10.1038/sj.npp.1301634. 18046309 PMC5688517

[ref16] Wilson-Poe A. R.; Jeong H. J.; Vaughan C. W. Chronic morphine reduces the readily releasable pool of GABA, a presynaptic mechanism of opioid tolerance. J. Physiol. 2017, 595, 6541–6555. 10.1113/JP274157. 28815604 PMC5638879

[ref17] Garzón J.; Rodríguez-Muñoz M.; Sánchez-Blázquez P. Direct association of Mu-opioid and NMDA glutamate receptors supports their cross-regulation: molecular implications for opioid tolerance. Curr. Drug Abuse Rev. 2012, 5, 199–226. 10.2174/1874473711205030199. 22920535

[ref18] Chapman V.; Haley J. E.; Dickenson A. H. Electrophysiologic analysis of preemptive effects of spinal opioids on N-methyl-D-aspartate receptor-mediated events. Anesthesiology 1994, 81, 1429–1435. 10.1097/00000542-199412000-00018. 7992912

[ref19] Sigtermans M. J.; van Hilten J. J.; et al. Ketamine produces effective and long-term pain relief in patients with Complex Regional Pain Syndrome Type 1. Pain 2009, 145, 304–311. 10.1016/j.pain.2009.06.023. 19604642

[ref20] Traynelis S. F.; Wollmuth L. P.; et al. Glutamate receptor ion channels: Structure, regulation, and function. Pharmacol. Rev. 2010, 62, 405–496. 10.1124/pr.109.002451. 20716669 PMC2964903

[ref21] Okabe S.; Collin C.; et al. Hippocampal synaptic plasticity in mice overexpressing an embryonic subunit of the NMDA receptor. J. Neurosci. 1998, 18, 4177–4188. 10.1523/JNEUROSCI.18-11-04177.1998. 9592097 PMC6792823

[ref22] Bliss T. V. P.; Collingridge G. L. A synaptic model of memory: long-term potentiation in the hippocampus. Nature 1993, 361, 31–39. 10.1038/361031a0. 8421494

[ref23] Collingridge G. L.; Volianskis A.; et al. The NMDA receptor as a target for cognitive enhancement. Neuropharmacology 2013, 64, 13–26. 10.1016/j.neuropharm.2012.06.051. 22796429 PMC4696548

[ref24] Tang Y. P.; Shimizu E.; et al. Genetic enhancement of learning and memory in mice. Nature 1999, 401, 63–69. 10.1038/43432. 10485705

[ref25] Collingridge G. The role of NMDA receptors in learning and memory. Nature 1987, 330, 604–605. 10.1038/330604a0. 2825035

[ref26] Rezvani A. H. Involvement of the NMDA System in Learning and Memory. Anim. Model. Cogn. Impair. 2006, 37–48. 10.1201/9781420004335.ch4. 21204373

[ref27] Kleckner N. W.; Dingledine R. Requirement for glycine in activation of NMDA-receptors expressed in Xenopus oocytes. Science 1988, 241, 835–837. 10.1126/science.2841759. 2841759

[ref28] Nowak L.; Bregestovski P.; Ascher P.; Herbet A.; Prochiantz A. Magnesium gates glutamate-activated channels in mouse central neurones. Nature 1984, 307, 462–465. 10.1038/307462a0. 6320006

[ref29] Mayer M. L.; Westbrook G. L.; Guthrie P. B. Voltage-dependent block by Mg2+ of NMDA responses in spinal cord neurones. Nature 1984, 309, 261–263. 10.1038/309261a0. 6325946

[ref30] Macdermott A. B.; Mayer M. L.; Westbrook G. L.; Smith S. J.; Barker J. L. NMDA-receptor activation increases cytoplasmic calcium concentration in cultured spinal cord neurones. Nature 1986, 321, 519–522. 10.1038/321519a0. 3012362

[ref31] Reisberg B.; Doody R.; et al. Memantine in moderate-to-severe Alzheimer’s disease. N. Engl. J. Med. 2003, 348, 1333–1341. 10.1056/NEJMoa013128. 12672860

[ref32] Milnerwood A. J.; Raymond L. A. Early synaptic pathophysiology in neurodegeneration: insights from Huntington’s disease. Trends Neurosci. 2010, 33, 513–523. 10.1016/j.tins.2010.08.002. 20850189

[ref33] Hallett P. J.; Standaert D. G. Rationale for and use of NMDA receptor antagonists in Parkinson’s disease. Pharmacol. Ther. 2004, 102, 155–174. 10.1016/j.pharmthera.2004.04.001. 15163596

[ref34] Coyle J. T. NMDA receptor and schizophrenia: a brief history. Schizophr. Bull. 2012, 38, 920–926. 10.1093/schbul/sbs076. 22987850 PMC3446237

[ref35] Olney J. W.; Newcomer J. W.; Farber N. B. NMDA receptor hypofunction model of schizophrenia. J. Psychiatr. Res. 1999, 33, 523–533. 10.1016/S0022-3956(99)00029-1. 10628529

[ref36] XiangWei W.; Jiang Y.; Yuan H. De Novo Mutations and Rare Variants Occurring in NMDA Receptors. Curr. Opin. Physiol. 2018, 2, 27 10.1016/j.cophys.2017.12.013. 29756080 PMC5945193

[ref37] Park C. K.; Nehls D. G.; Graham D. I.; Teasdale G. M.; McCulloch J. The glutamate antagonist MK-801 reduces focal ischemic brain damage in the rat. Ann. Neurol. 1988, 24, 543–551. 10.1002/ana.410240411. 2853604

[ref38] Simon R. P.; Swan J. H.; Griffiths T.; Meldrum B. S. Blockade of N-methyl-D-aspartate receptors may protect against ischemic damage in the brain. Science 1984, 226, 850–852. 10.1126/science.6093256. 6093256

[ref39] Morikawa E.; Mori H.; et al. Attenuation of focal ischemic brain injury in mice deficient in the epsilon1 (NR2A) subunit of NMDA receptor. J. Neurosci. 1998, 18, 9727–9732. 10.1523/JNEUROSCI.18-23-09727.1998. 9822733 PMC6793323

[ref40] Berman R. M.; Cappiello A.; et al. Antidepressant effects of ketamine in depressed patients. Biol. Psychiatry 2000, 47, 351–354. 10.1016/s0006-3223(99)00230-9. 10686270

[ref41] aan het Rot M.; Collins K. A.; et al. Safety and Efficacy of Repeated-Dose Intravenous Ketamine for Treatment-Resistant Depression. Biol. Psychiatry 2010, 67, 139–145. 10.1016/j.biopsych.2009.08.038. 19897179

[ref42] Wu L.-J. J.; Zhuo M. Targeting the NMDA receptor subunit NR2B for the treatment of neuropathic pain. Neurotherapeutics 2009, 6, 693–702. 10.1016/j.nurt.2009.07.008. 19789073 PMC5084290

[ref43] Chen B. S.; Roche K. W. Regulation of NMDA receptors by phosphorylation. Neuropharmacology 2007, 53, 362–368. 10.1016/j.neuropharm.2007.05.018. 17644144 PMC2001266

[ref44] Xu L.; Pan Y.; Zhu Q.; Gong S.; Tao J.; Xu G.-Y.; Jiang X. Arcuate Src activation-induced phosphorylation of NR2B NMDA subunit contributes to inflammatory pain in rats. J. Neurophysiol. 2012, 108, 3024–3033. 10.1152/jn.01047.2011. 22993256

[ref45] Salter M. W.; Kalia L. V. Src kinases: a hub for NMDA receptor regulation. Nat. Rev. Neurosci. 2004, 5, 317–328. 10.1038/nrn1368. 15034556

[ref46] Ali D. W.; Salter M. W. NMDA receptor regulation by Src kinase signalling in excitatory synaptic transmission and plasticity. Curr. Opin. Neurobiol. 2001, 11, 336–342. 10.1016/S0959-4388(00)00216-6. 11399432

[ref47] Dawson V. L.; Dawson T. M.; London E. D.; Bredt D. S.; Snyder S. H. Nitric oxide mediates glutamate neurotoxicity in primary cortical cultures. Proc. Natl. Acad. Sci. U.S.A. 1991, 88, 6368–6371. 10.1073/pnas.88.14.6368. 1648740 PMC52084

[ref48] He L.; Fong J.; Von Zastrow M.; Whistler J. L. Regulation of opioid receptor trafficking and morphine tolerance by receptor oligomerization. Cell 2002, 108, 271–282. 10.1016/S0092-8674(02)00613-X. 11832216

[ref49] Gainetdinov R. R.; Premont R. T.; Bohn L. M.; Lefkowitz R. J.; Caron M. G. Desensitization of G protein-coupled receptors and neuronal functions. Annu. Rev. Neurosci. 2004, 27, 107–144. 10.1146/annurev.neuro.27.070203.144206. 15217328

[ref50] Wang Z.; Arden J.; Sadée W. Basal phosphorylation of mu opioid receptor is agonist modulated and Ca2+-dependent. FEBS Lett. 1996, 387, 53–57. 10.1016/0014-5793(96)00467-X. 8654566

[ref51] Fedele E.; Raiteri M. In vivo studies of the cerebral glutamate receptor/NO/cGMP pathway. Prog. Neurobiol. 1999, 58, 89–120. 10.1016/S0301-0082(98)00077-X. 10321798

[ref52] Chizh B. A.; Headley P. M.; Tzschentke T. M. NMDA receptor antagonists as analgesics: Focus on the NR2B subtype. Trends Pharmacol. Sci. 2001, 22, 636–642. 10.1016/S0165-6147(00)01863-0. 11730974

[ref53] Petrenko A. B.; Yamakura T.; Baba H.; Shimoji K. The Role of N-Methyl-d-Aspartate (NMDA) Receptors in Pain: A Review. Anesth. Analg. 2003, 1108–1116. 10.1213/01.ANE.0000081061.12235.55. 14500166

[ref54] Brown A. G. The dorsal horn of the spinal cord. Q. J. Exp. Physiol. 1982, 10.1113/expphysiol.1982.sp002630. 6281848

[ref55] Todd A. J. Neuronal circuitry for pain processing in the dorsal horn. Nat. Rev. Neurosci. 2010, 11, 823–836. 10.1038/nrn2947. 21068766 PMC3277941

[ref56] Boyce S.; Wyatt A.; et al. Selective NMDA NR2B antagonists induce antinociception without motor dysfunction: Correlation with restricted localisation of NR2B subunit in dorsal horn. Neuropharmacology 1999, 38, 611–623. 10.1016/S0028-3908(98)00218-4. 10340299

[ref57] Temi S.; Rudyk C.; Armstrong J.; Landrigan J. A.; Dedek C.; Salmaso N.; Hildebrand M. E. Differential expression of GluN2 NMDA receptor subunits in the dorsal horn of male and female rats. Channels (Austin) 2021, 15, 179–192. 10.1080/19336950.2020.1871205. PMC7849732 33509021

[ref58] Monyer H.; Burnashev N.; Laurie D. J.; Sakmann B.; Seeburg P. H. Developmental and regional expression in the rat brain and functional properties of four NMDA receptors. Neuron 1994, 12, 529–540. 10.1016/0896-6273(94)90210-0. 7512349

[ref59] Trujillo K. A.; Akil H. Inhibition of morphine tolerance and dependence by the NMDA receptor antagonist MK-801. Science 1991, 251, 85–87. 10.1126/science.1824728. 1824728

[ref60] Bernardi M.; Bertolini A.; Szczawinska K.; Genedani S. Blockade of the polyamine site of NMDA receptors produces antinociception and enhances the effect of morphine, in mice. Eur. J. Pharmacol. 1996, 298, 51–55. 10.1016/0014-2999(95)00778-4. 8867919

[ref61] Mao J.; Price D. D.; Caruso F. S.; Mayer D. J. Oral administration of dextromethorphan prevents the development of morphine tolerance and dependence in rats. Pain 1996, 67, 361–368. 10.1016/0304-3959(96)03120-X. 8951930

[ref62] Ruppa K. B.; King D.; Olson R. E. NMDA Antagonists of GluN2B Subtype and Modulators of GluN2A, GluN2C, and GluN2D Subtypes-Recent Results and Developments. Annu. Rep. Med. Chem. 2012, 89–103. 10.1016/B978-0-12-396492-2.00007-2.

[ref63] Wang H.; James M. L.; et al. pH-Sensitive NMDA inhibitors improve outcome in a murine model of SAH. Neurocrit. Care 2014, 20, 119–131. 10.1007/s12028-013-9944-9. 24420693

[ref64] Myers S. J.; Ruppa K. P.; et al. A Glutamate N-Methyl-d-Aspartate (NMDA) Receptor Subunit 2B-Selective Inhibitor of NMDA Receptor Function with Enhanced Potency at Acidic pH and Oral Bioavailability for Clinical Use. J. Pharmacol. Exp. Ther. 2021, 379, 41–52. 10.1124/jpet.120.000370. 34493631 PMC8626636

[ref65] Tahirovic Y. A.; Geballe M.; et al. Enantiomeric propanolamines as selective N-methyl-D-aspartate 2B receptor antagonists. J. Med. Chem. 2008, 51, 5506–5521. 10.1021/jm8002153. 18800760 PMC3142473

[ref66] Yuan H.; Myers S. J.; et al. Context-Dependent GluN2B-Selective Inhibitors of NMDA Receptor Function are Neuroprotective with Minimal Side Effects. Neuron 2015, 85, 1305 10.1016/j.neuron.2015.02.008. 25728572 PMC4368485

[ref67] Regan M. C.; Zhu Z.; et al. Structural elements of a pH-sensitive inhibitor binding site in NMDA receptors. Nat. Commun. 2019, 10, 321 10.1038/s41467-019-08291-1. 30659174 PMC6338780

[ref68] Karakas E.; Furukawa H. Crystal structure of a heterotetrameric NMDA receptor ion channel. Science 2014, 344, 992–997. 10.1126/science.1251915. 24876489 PMC4113085

[ref69] Stroebel D.; Buhl D. H.; et al. A Novel Binding Mode Reveals Two Distinct Classes of NMDA Receptor GluN2B-selective Antagonists. Mol. Pharmacol. 2016, 89, 541–551. 10.1124/mol.115.103036. 26912815 PMC4859819

[ref70] Sheng M.; Cummings J.; Roldan L. A.; Jan Y. N.; Jan L. Y. Changing subunit composition of heteromeric NMDA receptors during development of rat cortex. Nature 1994, 368, 144–147. 10.1038/368144a0. 8139656

[ref71] Chazot P. L.; Coleman S. K.; Cik M.; Stephenson F. A. Molecular characterization of N-methyl-D-aspartate receptors expressed in mammalian cells yields evidence for the coexistence of three subunit types within a discrete receptor molecule. J. Biol. Chem. 1994, 269, 24403–24409. 10.1016/S0021-9258(19)51098-5. 7929101

[ref72] Hansen K. B.; Ogden K. K.; Yuan H.; Traynelis S. F. Distinct functional and pharmacological properties of Triheteromeric GluN1/GluN2A/GluN2B NMDA receptors. Neuron 2014, 81, 1084–1096. 10.1016/j.neuron.2014.01.035. 24607230 PMC3957490

[ref73] Vicini S.; Wang J. F.; et al. Functional and pharmacological differences between recombinant N-methyl-D-aspartate receptors. J. Neurophysiol. 1998, 79, 555–566. 10.1152/jn.1998.79.2.555. 9463421

[ref74] Karakas E.; Simorowski N.; Furukawa H. Structure of the zinc-bound amino-terminal domain of the NMDA receptor NR2B subunit. EMBO J. 2009, 28, 3910–3920. 10.1038/emboj.2009.338. 19910922 PMC2797058

[ref75] Karakas E.; Simorowski N.; Furukawa H. Subunit arrangement and phenylethanolamine binding in GluN1/GluN2B NMDA receptors. Nature 2011, 475, 249–253. 10.1038/nature10180. 21677647 PMC3171209

[ref76] Lee C. H.; Lü W.; et al. NMDA receptor structures reveal subunit arrangement and pore architecture. Nature 2014, 511, 191–197. 10.1038/nature13548. 25008524 PMC4263351

[ref77] Chou T.-H.; Epstein M.; et al. Structural insights into binding of therapeutic channel blockers in NMDA receptors. Nat. Struct. Mol. Biol. 2022, 29, 507–518. 10.1038/s41594-022-00772-0. 35637422 PMC10075384

[ref78] Chou T.-H.; Tajima N.; Romero-Hernandez A.; Furukawa H. Structural Basis of Functional Transitions in Mammalian NMDA Receptors. Cell 2020, 182, 357–371.e13. 10.1016/j.cell.2020.05.052. 32610085 PMC8278726

[ref79] Tajima N.; Karakas E.; et al. Activation of NMDA receptors and the mechanism of inhibition by ifenprodil. Nature 2016, 534, 63–68. 10.1038/nature17679. 27135925 PMC5136294

[ref80] Sewell R. D. E.; Spencer P. S. J. Antinociceptive activitiy of narcotic agonist and partial agonist analgesics and other agents in the tail-immersion test in mice and rats. Neuropharmacology 1976, 15, 683–688. 10.1016/0028-3908(76)90037-X. 12485

[ref81] Luttinger D. Determination of antinociceptive efficacy of drugs in mice using different water temperatures in a tail-immersion test. J. Pharmacol. Methods 1985, 13, 351–357. 10.1016/0160-5402(85)90017-8. 3927065

[ref82] Wong C. S.; Cherng C. H.; Luk H. N.; Ho S. T.; Tung C. S. Effects of NMDA receptor antagonists on inhibition of morphine tolerance in rats: binding at mu-opioid receptors. Eur. J. Pharmacol. 1996, 297, 27–33. 10.1016/0014-2999(95)00728-8. 8851162

[ref83] Pantouli F.; Grim T. W.; et al. Comparison of morphine, oxycodone and the biased MOR agonist SR-17018 for tolerance and efficacy in mouse models of pain. Neuropharmacology 2021, 185, 108439 10.1016/j.neuropharm.2020.108439. 33345829 PMC7887086

[ref84] Ellison G. The N-methyl-d-aspartate antagonists phencyclidine, ketamine and dizocilpine as both behavioral and anatomical models of the dementias. Brain Res. Rev. 1995, 20, 250–267. 10.1016/0165-0173(94)00014-G. 7795658

[ref85] Andiné P.; Widermark N.; et al. Characterization of MK-801-Induced Behavior as a Putative Rat Model of Psychosis. J. Pharmacol. Exp. Ther. 1999, 1, 1393–1408. 10454519

[ref86] Tricklebank M. D.; Singh L.; Oles R. J.; Preston C.; Iversen S. D. The behavioural effects of MK-801: a comparison with antagonists acting non-competitively and competitively at the NMDA receptor. Eur. J. Pharmacol. 1989, 167, 127–135. 10.1016/0014-2999(89)90754-1. 2550253

[ref87] Oliveira S. M.; Silva C. R.; et al. Antinociceptive effect of a novel armed spider peptide Tx3-5 in pathological pain models in mice. Pflugers Arch. - Eur. J. Physiol 2016, 468, 881–894. 10.1007/s00424-016-1801-1. 26898377

[ref88] Rigo F. K.; Trevisan G.; et al. Spider peptide Phα1β induces analgesic effect in a model of cancer pain. Cancer Sci. 2013, 104, 1226–1230. 10.1111/cas.12209. 23718272 PMC7657190

[ref89] Marshall I.; Weinstock M. Quantitative method for assessing one symptom of the withdrawal syndrome in mice after chronic morphine administration. Nature 1971, 234, 223–224. 10.1038/234223a0. 4943089

[ref90] Mccauley J. A.; Theberge C. R.; et al. NR2B-Selective N-Methyl-D-aspartate Antagonists: Synthesis and Evaluation of 5-Substituted Benzimidazoles. J. Med. Chem. 2004, 2089–2096. 10.1021/jm030483s. 15056006

[ref91] Mccool B. A.; Lovinger D. M. Ifenprodil inhibition of the 5-Hydroxytryptamine3 receptor. Neuropharmacology 1995, 34, 621–629. 10.1016/0028-3908(95)00030-A. 7566498

[ref92] Chenard B. L.; Shalaby I. A.; et al. Separation of alpha 1 adrenergic and N-methyl-D-aspartate antagonist activity in a series of ifenprodil compounds. J. Med. Chem 1991, 34, 3085–3090. 10.1021/jm00114a018. 1681106

[ref93] Nutt J. G.; Gunzler S. A.; et al. Effects of a NR2B selective NMDA glutamate antagonist, CP-101,606, on dyskinesia and Parkinsonism. Mov. Disord. 2008, 23, 1860–1866. 10.1002/mds.22169. 18759356 PMC3390310

[ref94] Mott D. D.; Doherty J. J.; et al. Phenylethanolamines inhibit NMDA receptors by enhancing proton inhibition. Nat. Neurosci. 1998, 1, 659–667. 10.1038/3661. 10196581

[ref95] Renaud H. J.; Cui J. Y.; Khan M.; Klaassen C. D. Tissue Distribution and Gender-Divergent Expression of 78 Cytochrome P450 mRNAs in Mice. Toxicol. Sci. 2011, 124, 261–277. 10.1093/toxsci/kfr240. 21920951 PMC3216415

[ref96] Hrycay E. G.; Bandiera S. M. Expression, function and regulation of mouse cytochrome P450 enzymes: comparison with human P450 enzymes. Curr. Drug Metab. 2009, 10, 1151–1183. 10.2174/138920009790820138. 20166999

[ref97] Waxman D. J.; Holloway M. G. Sex Differences in the Expression of Hepatic Drug Metabolizing Enzymes. Mol. Pharmacol. 2009, 76, 215 10.1124/mol.109.056705. 19483103 PMC2713118

[ref98] Kobayashi K.; Abe C.; et al. Gender Difference of Hepatic and Intestinal CYP3A4 in CYP3AHumanized Mice Generated by a Human Chromosome-engineering Technique. Drug Metab. Lett. 2017, 11, 60–67. 10.2174/1872312811666170404153804. 28393714

[ref99] Smith J. C. A Review of Strain and Sex Differences in Response to Pain and Analgesia in Mice. Comp. Med. 2019, 69, 490 10.30802/AALAS-CM-19-000066. 31822324 PMC6935701

[ref100] Dahan A.; Kest B.; Waxman A. R.; Sarton E. Sex-specific responses to opiates: animal and human studies. Anesth. Analg. 2008, 107, 83–95. 10.1213/ane.0b013e31816a66a4. 18635471

[ref101] Cicero T. J.; Nock B.; Meyer E. R. Gender-related differences in the antinociceptive properties of morphine. J. Pharmacol. Exp. Ther. 1996, 279, 695–701. 10.1124/jpet.300.2.695. 8930182

[ref102] Cook C. D.; Barrett A. C.; Roach E. L.; Bowman J. R.; Picker M. J. Sex-related differences in the antinociceptive effects of opioids: importance of rat genotype, nociceptive stimulus intensity, and efficacy at the μ opioid receptor. Psychopharmacology 2000, 150, 430–442. 10.1007/s002130000453. 10958085

[ref103] Dedek A.; Xu J.; et al. Sexual dimorphism in a neuronal mechanism of spinal hyperexcitability across rodent and human models of pathological pain. Brain 2022, 145, 1124 10.1093/brain/awab408. 35323848 PMC9050559

[ref104] Wiesenfeld-Hallin Z. Sex differences in pain perception. Gender Med. 2005, 2, 137–145. 10.1016/S1550-8579(05)80042-7. 16290886

[ref105] Craft R. M.; Lee D. A. NMDA antagonist modulation of morphine antinociception in female vs. male rats. Pharmacol. Biochem. Behav. 2005, 80, 639–649. 10.1016/j.pbb.2005.02.003. 15820534

[ref106] Gabel F.; Hovhannisyan V.; Andry V.; Goumon Y. Central metabolism as a potential origin of sex differences in morphine antinociception but not induction of antinociceptive tolerance in mice. Br. J. Pharmacol. 2022, 10.1111/bph.15792. 34986502

[ref107] Smith H. S. Opioid Metabolism REVIEW. Mayo Clin. Proc. 2009, 84, 613–624. 10.1016/S0025-6196(11)60750-7. 19567715 PMC2704133

[ref108] De Gregori S.; De Gregori M.; et al. Morphine metabolism, transport and brain disposition. Metab. Brain Dis. 2012, 27, 1 10.1007/s11011-011-9274-6. 22193538 PMC3276770

[ref109] Buckley D. B.; Klaassen C. D. Tissue- and Gender-Specific mRNA Expression of UDP-Glucuronosyltransferases (UGTs) in Mice. Drug Metab. Dispos. 2007, 35, 121–127. 10.1124/dmd.106.012070. 17050650

[ref110] Kitaori K.; Furukawa Y.; Yoshimoto H.; Otera J. CsF in organic synthesis. Regioselective nucleophilic reactions of phenols with oxiranes leading to enantiopure β-blockers. Tetrahedron 1999, 55, 14381–14390. 10.1016/S0040-4020(99)00896-0.

[ref111] Sajiki H.; Hattori K.; Hirota K. Highly chemoselective hydrogenation with retention of the epoxide function using a heterogeneous Pd/C - Ethylenediamine catalyst and THF. Chem. - Eur. J. 2000, 10.1002/1521-3765(20000616)6:12<2200::AID-CHEM2200>3.0.CO;2-3. 10926226

[ref112] Traynelis S. F.; Burgess M. F.; Zheng F.; Lyuboslavsky P.; Powers J. L. Control of voltage-independent Zinc inhibition of NMDA receptors by the NR1 subunit. J. Neurosci. 1998, 18, 6163–6175. 10.1523/jneurosci.18-16-06163.1998. 9698310 PMC6793196

[ref113] Jackson M. R.; Nilsson T.; Peterson P. A. Retrieval of transmembrane proteins to the endoplasmic reticulum. J. Cell Biol. 1993, 121, 317–333. 10.1083/jcb.121.2.317. 8468349 PMC2200111

[ref114] Jackson M. R.; Nilsson T.; Peterson P. A. Identification of a consensus motif for retention of transmembrane proteins in the endoplasmic reticulum. EMBO J. 1990, 9, 3153–3162. 10.1002/j.1460-2075.1990.tb07513.x. 2120038 PMC552044

[ref115] Zerangue N.; Malan M. J.; et al. Analysis of endoplasmic reticulum trafficking signals by combinatorial screening in mammalian cells. Proc. Natl. Acad. Sci. U.S.A. 2001, 98, 2431–2436. 10.1073/pnas.051630198. 11226256 PMC30155

[ref118] D’amour F. E.; Smith D. L. A method for determining loss of pain sensation. J. Pharmacol. Exp. Ther. 1941, 72, 74–79.

[ref119] Ben-Bassat J.; Peretz E.; Sulman F. G. Analgesimetry and ranking of analgesic drugs by the receptacle method. Arch. Int. Pharmacodyn. Ther. 1959, 122, 434–447. 13798682

[ref120] Janssen P. A.; Niemegeers C. J.; Dony J. G. The inhibitory effect of fentanyl and other morphine-like analgesics on the warm water induced tail withdrawl reflex in rats. Arzneimittelforschung 1963, 13, 502–507. 13957426

[ref121] Grotto M.; Sulman F. G. Modified receptacle method for animal analgesimetry. Arch. Int. Pharmacodyn. Ther. 1967, 165, 152–159. 5339935

[ref122] Faul F.; Erdfelder E.; Lang A. G.; Buchner A. G*Power 3: a flexible statistical power analysis program for the social, behavioral, and biomedical sciences. Behav. Res. Methods 2007, 39, 175–191. 10.3758/BF03193146. 17695343

[ref123] Besnard J.; Ruda G. F.; et al. Automated design of ligands to polypharmacological profiles. Nature 2012, 492, 215–220. 10.1038/nature11691. 23235874 PMC3653568

