# Supplementary material for: Novel GluN2B-Selective NMDA Receptor Negative Allosteric Modulator Possesses Intrinsic Analgesic Properties and Enhances Analgesia of Morphine in a Rodent Tail Flick Pain Model
Source: ACS Chem Neurosci. 2023 Feb 13;14(5):917–35. doi: 10.1021/acschemneuro.2c00779 (PMC9983021; doi:10.1021/acschemneuro.2c00779)
Supplement: Supplementary file 1 — cn2c00779_si_001.pdf [file cn2c00779_si_001.pdf]

Novel GluN2B-Selective NMDA Receptor Negative Allosteric Modulator Possesses Intrinsic Analgesic Properties and Enhances Analgesia of Morphine in a Rodent Tail Flick Pain Model

Lynnea D. Harris[1], Michael C. Regan[3,4], Scott J. Myers[2], Kelsey A. Nocilla[2], Nicholas S. Akins[1], Yesim A. Tahirovic[1], Lawrence J. Wilson[1], Ray Dingledine[2], Hiro Furukawa[3], Stephen F. Traynelis[2]*, Dennis C. Liotta[1]

[1]Department of Chemistry, Emory University, Atlanta, GA, USA 30322

[2]Department of Pharmacology and Chemical Biology, Emory University, Atlanta, GA, USA 30322

[3]W.M. Keck Structural Biology Laboratory, Cold Spring Harbor Laboratory, New York, NY, USA 11724

[4] RADD Pharmaceuticals, Westport, CT, USA 06880

*Corresponding Author

Supporting Information for Publication

Supplemental Table S1. Table of EU93-108 concentration-inhibition results at NMDA receptors expressed in *Xenopus* oocytes. Receptor subtypes shown correspond to receptor subtypes and data presented in Figure 2. * indicates a significant difference from r2Bc1/r2Bc2 and h2B/h2B receptors by one-way ANOVA and Tukey's multiple comparison test, $p<0.05$ or better. Statistical tests were conducted on the LogIC_{50} values. N represents the number of oocytes evaluated.

Receptor	IC_{50} μM	IC_{50} μM 95% CI	nH	nH 95% CI	Ymin	95% CI	N
**r2Ac1/r2Ac2**	ND	ND	ND	ND	104.8	(102, 107.5)	6
**r2Ac1/r2Bc2**	0.543*	(0.460, 0.640)	-0.92*	(-1.0, -0.85)	54.0*	(50.3, 57.7)	12
**r2Bc1/r2Bc2**	0.233	(0.196, 0.279)	-1.06	(-1.09, -1.02)	10.6	(7.9, 13.3)	8
**h2B/h2B**	0.261	(0.197, 0.347)	-1.16	(-1.25, -1.07)	12.0	(8.5, 15.5)	10

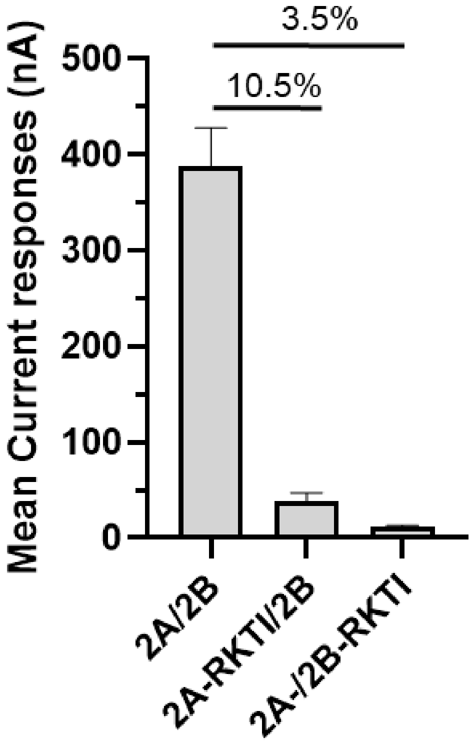

Supplemental Figure S1. The mean raw current ± SEM in nanoamperes (nA) in response to exposure of *Xenopus* oocytes recorded under two electrode voltage clamp to 100 μM glutamate and 100 μM glycine. Values for wild type (2A/2B) or variant GluN1/GluN2A$_{C1}$/GluN2B$_{C2}$ triheteromeric NMDARs are shown that have a wild type agonist binding domain or harbor the RKTI mutations in the agonist binding domain that renders the indicated GluN2 subunit incapable of binding glutamate (see Methods). The average nA current response for RKTI mutations is shown as the percentage of that observed for NMDARs with functional glutamate binding sites indicated at the top. This percentage is an estimate of the amount of current that could potentially reflect diheteromeric receptors that escape the ER retention strategy and reach the surface with two copies of either the GluN2$_{C1}$ or two copies of GluN2$_{C2}$ subunit (see Hansen et al., 2014).

Data Collection	ATD + EU93-108
Beamline	NSLS-II (17-ID-1)
Space group	C2
Wavelength (Å)	0.9198
Unit cell dimensions	
a, b, c (Å)	269.172
	60.076
	145.572
β (º)	117.020
Resolution (Å)	50.00-2.85 (2.90)
R_{merge}	0.073 (0.776)
I/σs	14.7 (1.0)
Completeness (%)	99.1 (91.2)
Redundancy	3.3 (2.3)
**Refinement**	
Resolution (Å)	35 – 2.85
No. reflection	48,988
R_{work}/R_{free}	0.174/0.246
No. atoms	
Protein	11,244
Ligand	60
Na	2
Water	94
B factors (Å^2)	
Protein	57.01
Ligand	54.40
Na	44.79
Water	39.00
R.M.S. deviations	
Bond length (Å)	0.007
Bond angles (º)	1.527

Supplemental Table S2. X-ray crystallographic data collection and model refinement statistics. All datasets were collected from a single crystal. Values in parentheses are for the highest-resolution shell.

Methods for Chung Spinal Nerve Ligation

The Chung spinal nerve ligation model was implemented by Algos Therapeutics. Male Sprague-Dawley rats (Hsd:Sprague-Dawley®™SD®™, Harlan, Indianapolis, Indiana, U.S.A.) weighing 222 ± 1 g were housed three per cage and given *ad libitum* access to food and water. Animal holding rooms operated on a 12:12h light/dark schedule for the entire duration of the study. The animal colony was maintained at 21ºC and 60% humidity. All experiments were conducted in accordance with the International Association for the Study of Pain guidelines and were approved by the University of Minnesota Animal Care and Use Committee.

Allodynia was induced via the Chung spinal nerve ligation (SNL) method[116] in which the animals were anesthetized with isoflurane, the left L5 transverse process was removed, then the L5 and L6 spinal nerves were tightly ligated with 6-0 silk suture. Finally, the wound was closed with internal sutures and external staples. Allodynia was assessed using 8 Semmes-Weinstein filaments (Stoelting, Wood Dale, IL, USA) with varying stiffness (0.4, 0.7, 1.2, 2.0, 3.6, 5.5, 8.5, and 15 g) following the up-down method[117] first published by Chaplan et al. in 1994. Baseline measurements were taken two weeks following SNL and prior to compound administration. Measurements were also taken 30, 60, 120 and 240 minutes post intraperitoneal compound injection. Any animals displaying lethargy were excluded from the study.

Supporting References for Chung Spinal Nerve Ligation

116. Ho Kim, S. & Mo Chung, J. An experimental model for peripheral neuropathy produced by segmental spinal nerve ligation in the rat. Pain 50, 355–363 (1992).

117. Chaplan, S. R., Bach, F. W., Pogrel, J. W., Chung, J. M. & Yaksh, T. L. Quantitative assessment of tactile allodynia in the rat paw. J. Neurosci. Methods 53, 55–63 (1994).

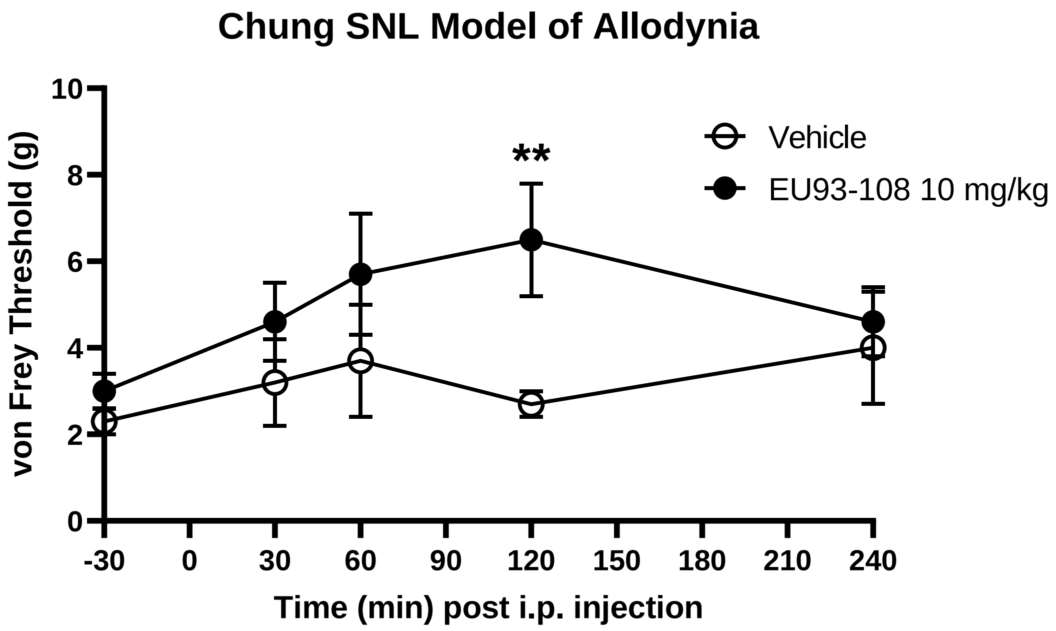

Supplemental Figure S2. EU93-108 is efficacious in the Chung spinal nerve ligation model of allodynia in male Sprague-Dawley rats. The allodynia data compares vehicle (5% DMSO, 50% PEG, 5% DMA in water, open circles) with 10 mg/kg EU93-108 (closed circles). Von Frey testing took place 30 minutes prior to injection and 30-, 60-, 120-, and 240-minutes post injection. Each symbol indicates n=10 rats.

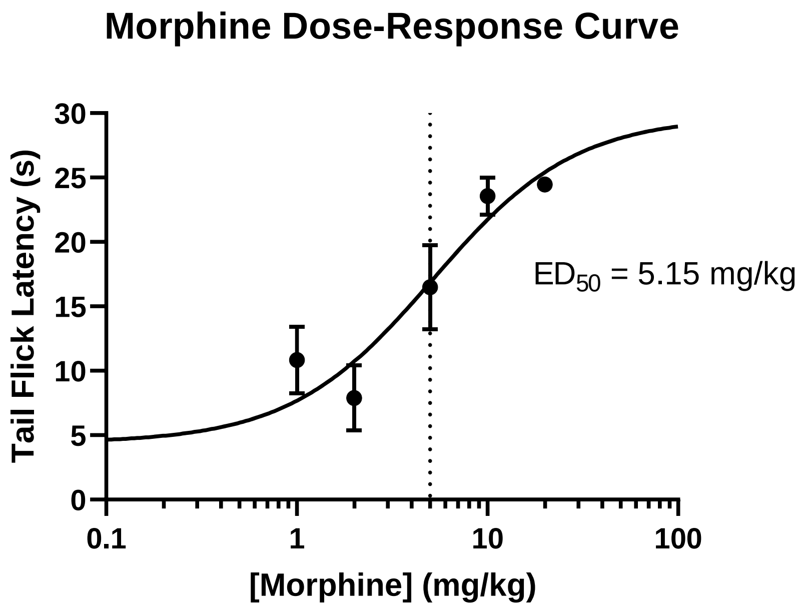

Supplemental Figure S3. Morphine dose-response curve in male C57BL/6J mice. Morphine was given s.c. at 1, 2, 5, 10 and 20 mg/kg (N=8 mice per dose). Tail immersion tests were conducted 30 minutes post injection. A 25-second cut-off time was implemented for each experiment. Each circle represents the mean ± SEM for each dose. The dotted line depicts the estimated ED_{50} value.

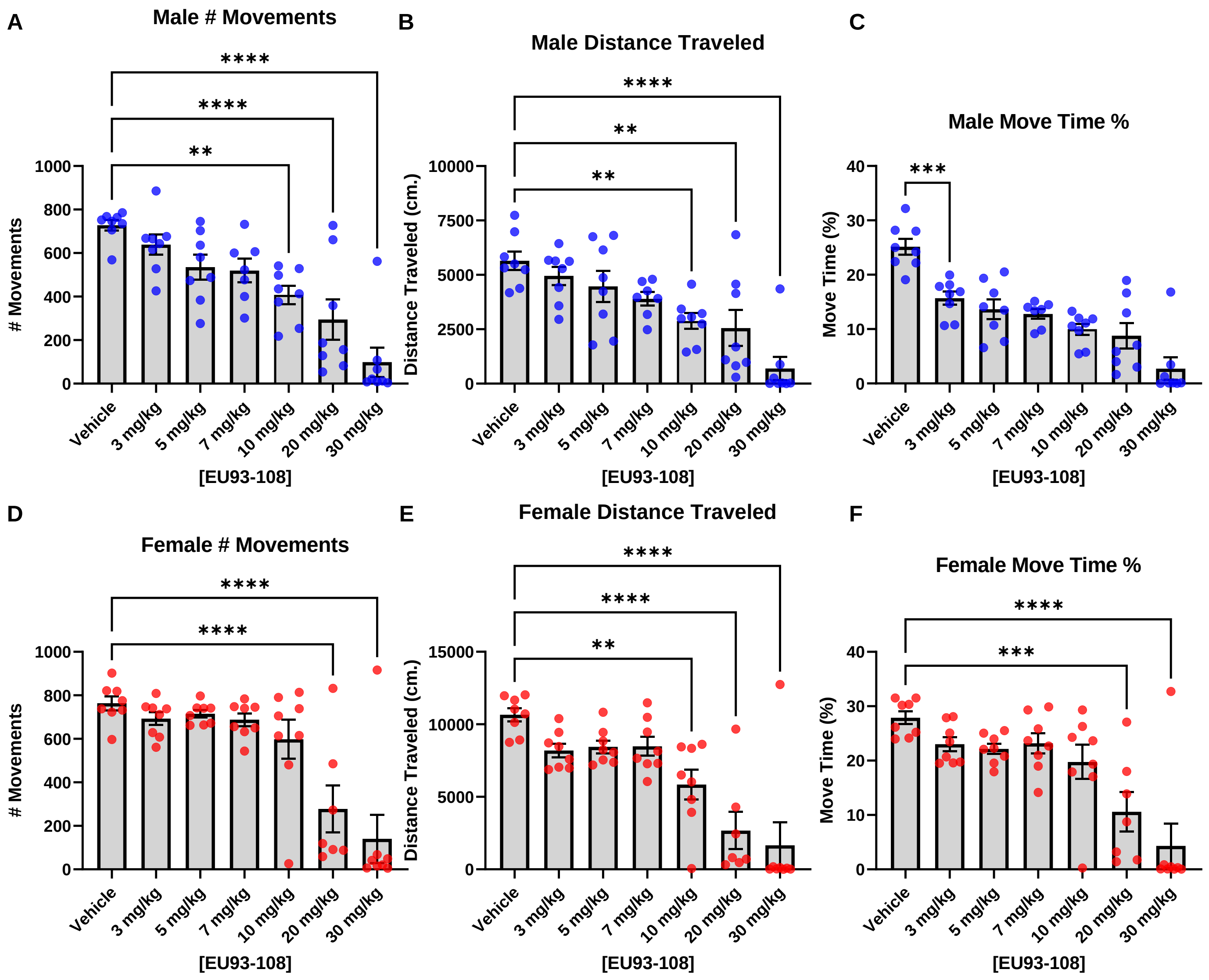

Supplemental Figure S4. Locomotor activity of EU93-108. Data for male mice are shown in panels A-C, and data for female mice shown in panels D-F. (A) and (D) depict the number of movements made in the locomotor box during the one-hour experiment. (B) and (E) depict the average distance traveled in centimeters. (C) and (F) depict the average percentage of time the mice spent moving. All data are presented as mean ± SEM. Each dot represents one mouse, N=8 mice per group. Data were analyzed using a one-way ANOVA and Dunnett's post hoc test for multiple comparisons, where each group was compared to vehicle. P < 0.5 constitutes significance.

Receptor	Agonist	% Control<br>10 μM EU93-108
**GluN1/GluN2A**	100 *μ*M glutamate,<br>30 *μ*M glycine	95.6 ± 1.6 (7)
**GluN1/GluN2B**	100 *μ*M glutamate,<br>30 *μ*M glycine	13.2 ± 6.1 (8)
**GluN1/GluN2C**	100 *μ*M glutamate,<br>30 *μ*M glycine	97 ± 2.8 (7)
**GluN1/GluN2D**	100 *μ*M glutamate,<br>30 *μ*M glycine	97 ± 1.9 (6)
**GluA1**	100 *μ*M glutamate	101.3 ± 1.9 (8)
**GluA2-R607Q**	100 *μ*M glutamate	98.4 ± 0.57 (8)
**hGluA3-L531Y**	100 *μ*M glutamate	94.7 ± 5.1 (8)
**GluK2**	100 *μ*M glutamate	88.1 ± 4.4 (8)
**hGluN1/GluN3A**	100 *μ*M glycine	103.6 ± 1.7 (9)
**rGluN1/GluN3B**	100 *μ*M glycine	108.8 ± 4.0 (8)
**α4β2-nACh**	10 *μ*M acetylcholine	3.2 ± 0.7 (8)
**α7-nACh**	300 *μ*M acetylcholine	70.9 ± 6.5 (7)
**5-HT$_{3A}$**	100 *μ*M serotonin	92.8 ± 2.7 (8)
**α1β2γ2S-GABA$_A$**	100 *μ*M GABA	92.8 ± 1.5 (9)
**ρ-GABA$_C$**	100 *μ*M GABA	97 ± 1.5 (5)
**α1-Glycine**	100 *μ*M glycine	105 ± 6.6 (8)
**hP2x**	9 *μ*M ATP	96.6 ± 4.3 (11)

Supplemental Table S3. Off-target actions of EU93-108 at ligand-gated ion channels expressed in *Xenopus* oocytes. The mean ± standard error of the mean for agonist plus 10 μM EU93-108 as a percentage of agonist in vehicle are given for each compound and receptor tested. Number of oocytes tested is given in parentheses.

Receptor	Mean % Inhibition	Receptor	Mean % Inhibition	Receptor	Mean % Inhibition
5-HT_{1A}	22.81	$Alpha_{2B}$	67.97	H_2	13.66
5-HT_{1B}	-7.29	$Alpha_{2C}$	62.97	H_3	22.66
5-HT_{1D}	39.2	$Beta_1$	29.14	H_4	2.12
5-HT_{1E}	1.68	$Beta_2$	13.7	KOR	-3.1
5-HT_{2A}	83.75	$Beta_3$	-10.49	M_1	-18.06
5-HT_{2B}	67.36	BZP Rat Brain Site	14.68	M_2	-23.09
5-HT_{2C}	71.28	D_1	24.67	M_3	9.26
5-HT_3	0.87	D_2	23.12	M_4	51.36
5-HT_{5A}	12.99	D_3	67.14	M_5	-0.11
5-HT_6	25.27	D_4	11.37	MOR	4.04
5-HT_{7A}	55.89	D_5	8.94	NET	12.52
$Alpha_{1A}$	86.3	DAT	-7.01	PBR	-0.34
$Alpha_{1B}$	5.09	DOR	-8.82	SERT	15.1
$Alpha_{1D}$	76.65	$GABA_A$	1.91	$Sigma_1$	69.21
$Alpha_{2A}$	77.72	H_1	76.87	$Sigma_2$	47.16

Supplemental Table S4. Off-Target Actions of EU93-108: Primary GPCR Screen. Cloned human molecular targets were individually expressed and submitted to molecular target-based screening with initial screens performed at a final concentration of 10 µM in quadruplicate. Complete protocols for the assays have been previously published (Besnard J *et al*. 2012). Where inhibition greater than 50% was measured, secondary screens were performed wherein K_i values were calculated (see Table 4).
